# Molecular Basis of Orb2 Amyloidogenesis and Blockade of Memory Consolidation

**DOI:** 10.1371/journal.pbio.1002361

**Published:** 2016-01-26

**Authors:** Rubén Hervás, Liying Li, Amitabha Majumdar, María del Carmen Fernández-Ramírez, Jay R. Unruh, Brian D. Slaughter, Albert Galera-Prat, Elena Santana, Mari Suzuki, Yoshitaka Nagai, Marta Bruix, Sergio Casas-Tintó, Margarita Menéndez, Douglas V. Laurents, Kausik Si, Mariano Carrión-Vázquez

**Affiliations:** 1 Instituto Cajal, IC-CSIC, Madrid, Spain; 2 Instituto Madrileño de Estudios Avanzados en Nanociencia (IMDEA-Nanociencia), Madrid, Spain; 3 Stowers Institute for Medical Research, Kansas City, Missouri, United States of America; 4 Department of Molecular and Integrative Physiology, University of Kansas Medical Center, Kansas City, Kansas, United States of America; 5 National Brain Research Centre, Manesar, Guragon, Haryana, India; 6 Department of Degenerative Neurological Diseases, National Institute of Neuroscience, National Center of Neurology and Psychiatry, Kodaira, Tokyo, Japan; 7 Core Research for Evolutional Science and Technology (CREST), Japan Science and Technology Agency, Saitama, Japan; 8 Instituto de Química-Física Rocasolano, IQFR-CSIC, Madrid, Spain; 9 Centro de Investigación Biomédica en Red sobre Enfermedades Respiratorias, Madrid, Spain; Baylor College of Medicine, UNITED STATES

## Abstract

Amyloids are ordered protein aggregates that are typically associated with neurodegenerative diseases and cognitive impairment. By contrast, the amyloid-like state of the neuronal RNA binding protein Orb2 in *Drosophila* was recently implicated in memory consolidation, but it remains unclear what features of this functional amyloid-like protein give rise to such diametrically opposed behaviour. Here, using an array of biophysical, cell biological and behavioural assays we have characterized the structural features of Orb2 from the monomer to the amyloid state. Surprisingly, we find that Orb2 shares many structural traits with pathological amyloids, including the intermediate toxic oligomeric species, which can be sequestered in vivo in hetero-oligomers by pathological amyloids. However, unlike pathological amyloids, Orb2 rapidly forms amyloids and its toxic intermediates are extremely transient, indicating that kinetic parameters differentiate this functional amyloid from pathological amyloids. We also observed that a well-known anti-amyloidogenic peptide interferes with long-term memory in *Drosophila*. These results provide structural insights into how the amyloid-like state of the Orb2 protein can stabilize memory and be nontoxic. They also provide insight into how amyloid-based diseases may affect memory processes.

## Abstract 2

Although amyloids are usually associated with abnormality and disease, some are functional. A study of the functional amyloid Orb2, which is needed for memory consolidation in flies, shows that despite structural similarities, key features of the kinetics of amyloid formation may underlie this crucial difference.

## Author Summary

Amyloids are ordered protein aggregates typically associated with neurodegenerative diseases, such as Alzheimer disease and Parkinson disease, which usually result in cognitive impairment. However, the amyloid state of the neuronal RNA binding protein Orb2 shows a quite opposite behaviour: instead of impairment, it is associated with memory consolidation. Here, we have characterized the structural features of Orb2 from the monomer to the amyloid state in order to determine why it shows this unexplained puzzling behaviour. Surprisingly, we find that Orb2 shares many structural traits with pathological amyloids. However, Orb2 rapidly forms amyloids, and its toxic intermediates are transient. Furthermore, we find that an anti-amyloidogenic peptide that blocks the initial structural transitions in pathological amyloids also blocks Orb2 amyloid formation and memory consolidation. Our results provide structural insights into how Orb2 amyloids can mediate stable memory while being nontoxic and how amyloid-based diseases may affect memory processes. They also suggest a new pharmacological approach for blocking memory consolidation.

## Introduction

Amyloids, whose cross-*β* fold has been postulated as the ancestral protein fold [1,2], were initially associated with fatal neurodegenerative disorders [3,4]. However, more than a century after the identification of these “pathological amyloids,” a growing list of so-called “functional amyloids” that fulfils a wide variety of physiological functions has recently emerged [5]. The discovery of functional amyloids raises the question of what causes such a strikingly distinct behaviour to that observed in pathological amyloids. Indeed, it remains unclear what features are shared by functional and pathological amyloids and what determines whether a particular amyloid is functional rather than toxic.

One functional amyloid-like protein of particular interest is the cytoplasmic polyadenylation element binding protein (CPEB). The CPEB family of proteins regulates the translation of dormant mRNAs [6,7], and some members of this family are involved in synaptic plasticity and long-lasting memory [8,9]. The CPEB isoforms share a common C-terminal catalytic region (RNA-binding domain), but they differ in the N-terminal region. Surprisingly, the N-terminus of some CPEB isoforms in *Aplysia*, *Drosophila*, and mouse have features characteristic of self-sustaining amyloidogenic (prion-like) proteins [9–13]. For example, the neuronal specific isoform of *Aplysia* CPEB (ApCPEB) has a glutamine-asparagine (Q/N)-rich N-terminal domain, which resembles a yeast prion-like domain (PLD) [14], and it is predicted to have conformational flexibility [10]. Indeed, ApCPEB undergoes a conformational transition to a *β*-sheet-rich state similar to that undertaken by other prion-like proteins [15]. In sensory neurons, the neurotransmitter serotonin controls the prion-like switch from the monomeric form to the self-sustaining oligomeric state, which is important for the serotonin-induced increase in synaptic strength [16]. Accordingly, it has been postulated that the switch to the oligomeric and self-perpetuating state contributes to the long-term maintenance of synapse-specific changes [16], providing a molecular mechanism for the persistence of memory [10].

The *Drosophila melanogaster* orthologue of CPEB, Orb2, has two isoforms that are structurally similar to the ApCPEB isoform: Orb2A and Orb2B [17,18]. Both forms are expressed in the fly brain and they are required for long-term memory [11,17,19]. Orb2A, a synaptic protein, is scarce, and its availability is controlled by phosphorylation [19, 20]. This isoform has eight amino acid residues (YNKFVNFI) preceding the PLD, which are critical for both the efficiency as well as the nature of the amyloid-like oligomers being formed [11]. The longer Orb2B isoform, with a region of 162 residues preceding the PLD, appears to act as a canonical CPEB [21], regulating translation via its RNA-binding domain [18,21]. The PLD of Orb2A has a low Q/N content compared to ApCPEB (23.5% versus 48.1%), it acts in long-term memory independently of its RNA-binding domain [21], and its mutations prevent memory consolidation [11,17].

The nervous system is particularly susceptible to amyloid-driven diseases, some of which lead to severe cognitive deficits [22]. Currently, it remains unclear why some amyloids in general are detrimental for neurons, while a seemingly similar amyloid state of some neuronal CPEB proteins is critical for memory stabilization [9,11]. As with other biological conundrums, a structural/functional analysis of CPEB/Orb2 proteins may shed light on the features that distinguish functional from pathological amyloids, and it may also help us to understand the molecular basis of memory consolidation. Here, we have employed an array of in vitro (bulk and single-molecule biophysics as well as cell culture) and in vivo (*Drosophila* and yeast) techniques to characterize Orb2 amyloid both structurally and functionally. In addition to dissecting out the characteristics of the Q/N-rich PLD-containing region of Orb2, we have found that a known anti-amyloidogenic peptide inhibits some forms of memory consolidation in *Drosophila*. Furthermore, comparing with pathological amyloids, we found that although in solution Orb2 can form toxic oligomers, these toxic species rapidly progress to innocuous species. These transient Orb2 species are structurally similar to toxic Huntingtin aggregates and, when abundant, these two proteins form a heteromeric complex. These findings indicate that there are intrinsic structural constraints that prevent functional amyloids to dwell in the toxic conformation. These observations also provide clues as to how pathological amyloids may interfere with the function of those beneficial amyloids.

## Results

### Orb2 Forms Canonical Amyloids Very Efficiently

**Fig 1:**
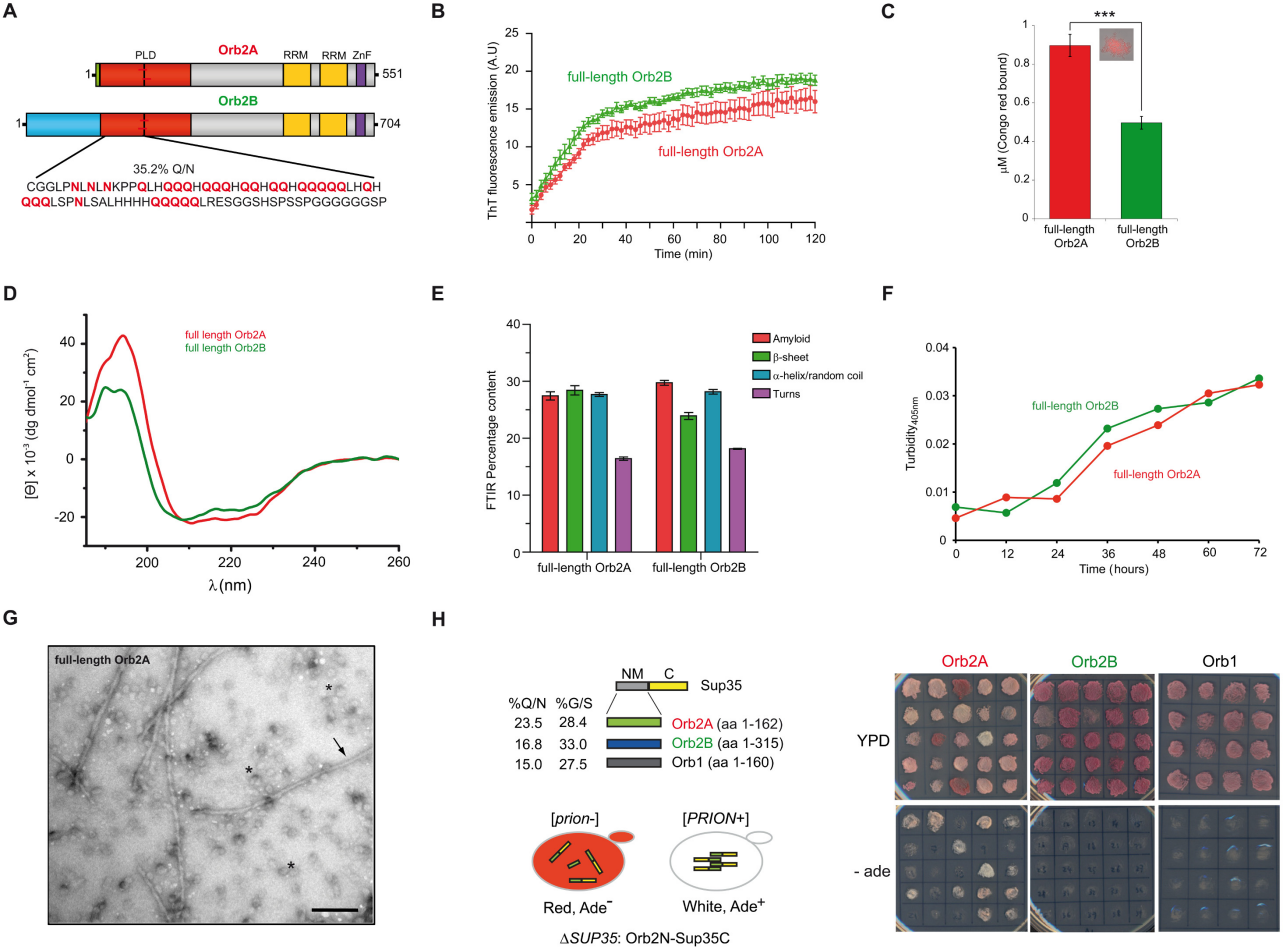
Orb2 forms self-propagating and canonical amyloids. **(A)** Pictograms showing the domain organization of Orb2A and Orb2B isoforms. PLD, prion-like domain, in red; RRM, RNA-binding domains, in yellow; ZnF, zinc-finger motif, in purple. The N-terminal amino acids preceding the PLD of Orb2A (eight amino acids) and Orb2B (162 amino acids) are represented in green and blue, respectively. **(B)** In the presence of both Orb2A and Orb2B, ThT (Proteostat) shows enhanced fluorescence emission at 485 nm over time, a typical feature of amyloids, although the kinetics is much faster for Orb2A and Orb2B than other amyloids (see S1 Fig). **(C)** Concentration (*μ*M) of the azo-dye CR bound to the amyloid component formed by Orb2. The data are represented as the mean ± standard error of the mean (SEM): ***p < 0.001 (One-way ANOVA and Tukey post-test). The inset shows a 63x image of an Orb2A amyloid under a polarized light microscope. **(D)** The CD spectra of soluble, full-length isoforms of Orb2 showed a α-helix-rich secondary structure. Deconvolution of the CD spectrum by using algorithms in DICHROWEB showed that Orb2A has 6% more α-helix and 5% less random coil conformations than Orb2B. **(E)** Relative abundance of secondary structural elements determined using FTIR. These distributions are significantly different from one another and from a non-amyloidogenic control protein concanavalin A control with a *p*-value of < 0.001 (chi-square test). **(F)** Aggregation of both isoforms over the incubation period monitored by turbidimetry at 405 nm. **(G)** Representative electron micrograph of oligomers (asterisks) and amyloid fibers (arrow) formed by Orb2A. Scale bar: 0.2 *μ*m. **(H)** Schematic representation of the constructs used in the yeast prion assay (left panel). Orb2A-Sup35C efficiently switches to the prion state. Randomly selected clones were replica plated either in complete yeast extract peptone dextrose (YPD) media or in media lacking adenine. Only Orb2A produced a high frequency of red Ade^-^ and white Ade^+^ colonies (right panel). NM: N-terminal and medial regions; C: C-terminal region. The underlying data for panels in this figure can be found in S1 Data.

Both the endogenous and the recombinant Orb2 protein form sodium dodecyl sulphate (SDS) and urea-resistant oligomers that are characteristic of amyloids [11]. However, it remains unclear to what extent the Orb2 amyloid behaves as a typical pathological amyloid. Both recombinant Orb2A and Orb2B bind to thioflavin T (ThT) and Congo red (CR) dyes (Fig 1A–1C and S1A Fig), and this binding is inhibited by amyloid destabilizing reagents such as rottlerin [23] or the polyphenol (-)-epigallocatechin gallate (EGCG: S1B Fig) [24,25]. The soluble form of both full-length isoforms has a helix-rich secondary structure monitored by Circular Dichroism (CD) spectroscopy (Fig 1D). However, over time both isoforms become rich in amyloid-specific *β*-sheets, as evidenced by Fourier transform infrared (FTIR) spectroscopy (Fig 1E), which coincides with aggregation of the protein, as monitored by turbidimetry (Fig 1F). Furthermore, transmission electron microscopy (TEM) showed Orb2A to form spherical oligomers and typical unbranched amyloid fibers (Fig 1G). Interestingly, Orb2A adopted an amyloid structure more efficiently (without the typical lag phase) than other amyloids like the A*β*42 peptide or the yeast prion Sup35NM, with a t½ of ~15 min and at a protein concentration 8–10-fold lower (S1C Fig). Based on these observations, we conclude that, in isolation, Orb2A not only forms seemingly canonical amyloid structures but it also does so in an extremely efficient manner.

### Prion-like Properties of Orb2A

In order to determine whether Orb2A amyloid possesses the self-sustaining properties of prion-like proteins, we took advantage of the well-characterized yeast prion Sup35, a translation termination factor that can exist in two states: as an active monomeric state (nonprion) and as a less active amyloid state (prion) [26]. The conversion between those two states can be readily assessed by non-sense suppression of the mutant *ade1-14* allele. In the nonprion state of Sup35, yeast colonies appear red in rich media, and they cannot grow in media containing a limiting amount of Adenine (-ade media), while in the prion-state, read-through translation turns the colonies white and cells can grow in -ade media. We substituted the NM prion domain of the Sup35 protein with the N-terminal domain of Orb2A, Orb2B or a paralogue of Orb2 in *Drosophila*, Orb1 (Fig 1H, left), and these chimeric constructs were expressed under the control of Sup35 promoter, representing the sole source of the Sup35 protein [27]. While all these chimeric proteins substituted the essential function of the Sup35 protein, only Sup35 carrying the N-terminal domain of Orb2A produced two readily distinguishable phenotypes: red Ade^-^ [*prion*-] and white Ade^+^ [*PRION*+] colonies (Fig 1H, right). The Orb2A N-terminal domain produced the prion-like state with an unusually high frequency and perpetuated stably through hundreds of generations. Random selection of 100 colonies revealed that almost 50% of them spontaneously gave rise to white-pink Ade^+^ colonies for Orb2A, compared to ~1% for Orb2B and none for Orb1 (Fig 1H, right). Furthermore, the nonprion and prion-like states correlated with the monomeric and SDS-urea resistant amyloidogenic oligomeric states of Orb2, respectively (S1D Fig). These results indicate Orb2A is very efficient in adopting a self-sustaining amyloid-like state.

### Boundaries and Conformational States of the Orb2A PLD

A group of the prion-like proteins characterized to date contain a PLD that is disordered and frequently composed of a Q/N rich sequence with a low content of charged residues [28–30]. In Orb2A, the entire PLD is composed of an N-terminal Q/N-rich domain (88 residues with 35.2%Q+N, Fig 1A), which is followed by a 74-residue region containing few charged residues (6.8% compared to 21.6% in the rest of the Orb2A protein).

**Fig 2:**
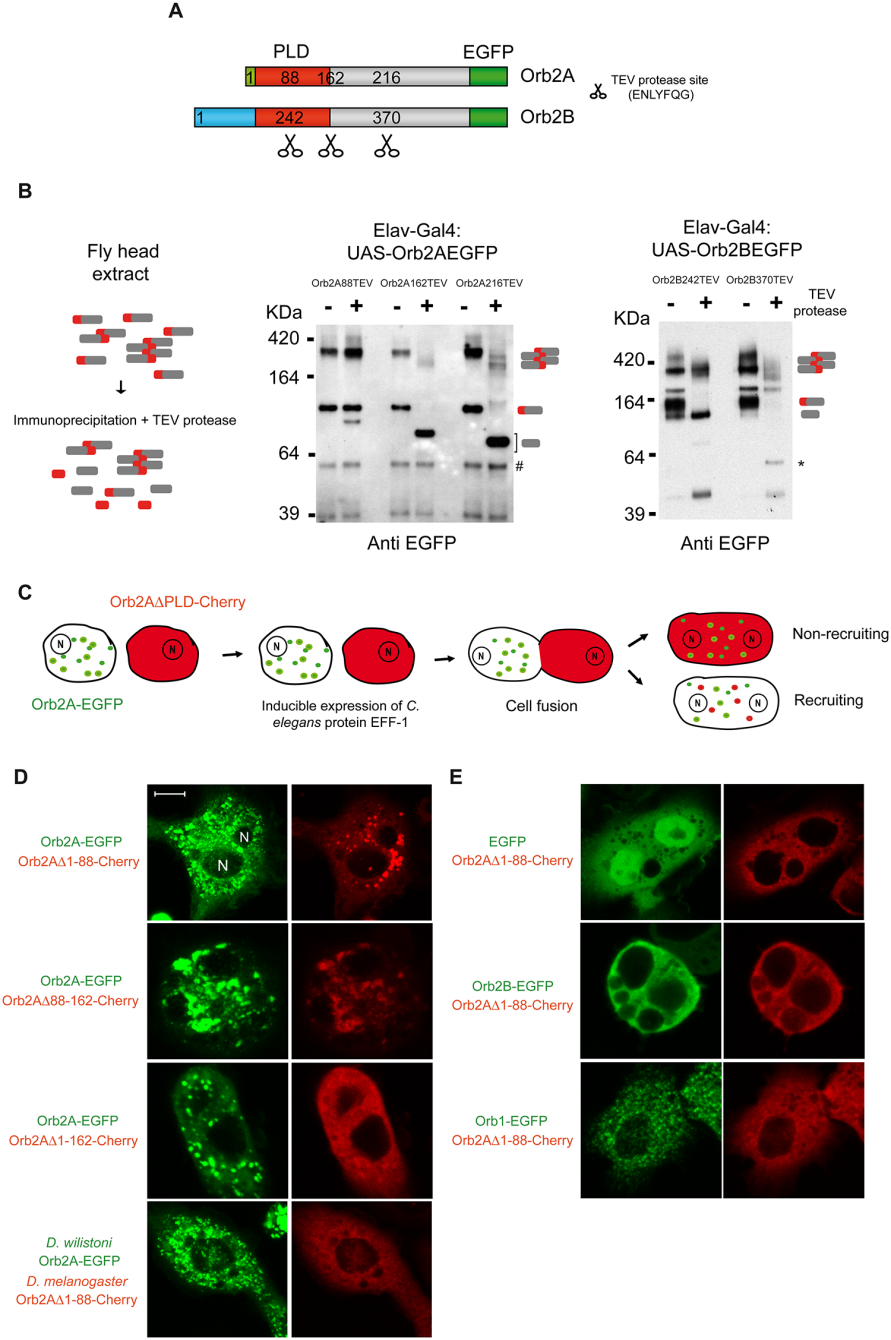
Organization of the Orb2 PLD. **(A)**. Schematic representation of the location of the TEV protease recognition motif (ENLYFQG) inserted into Orb2. **(B)** EGFP-tagged Orb2A and Orb2B bearing TEV protease sites were expressed pan-neuronally, and immunopurified Orb2 was treated with TEV protease for 24 h. The left panel shows the schematic of the experiment. The cleaved C-terminus of different sizes derived from Orb2A are indicated with a bracket. The * indicates an EGFP reactive polypeptide most likely originated from degradation of the cleaved Orb2B370TEV C-terminus. **(C)** Schematic diagram of the epithelial fusion failure 1 (EFF-1) cell fusion “cytoduction” experiment in S2 cells. The expected outcomes in terms of the recruitment or nonrecruitment of oligomers are indicated. **(D)** Residues 88 to 162 are sufficient for recruitment into pre-existing oligomers. Full-length Orb2A-EGFP induced the Orb2A construct lacking the 88 N-terminal residues (Orb2AΔ1-88-Cherry) to oligomerize. Full-length Orb2A from *D*. *willistoni* failed to induce oligomerization of Orb2AΔ1-88-Cherry. **(E)** EGFP, Orb2B-EGFP, or Orb1-EGFP also failed to induce oligomerization of Orb2AΔ1-88-Cherry. Representative examples of fused cells are shown and the *n* for each experimental set is ≥10. Scale bar = 5 *μ*m.

PLDs often adopt distinct conformational states, such as coiled-coil rich [31] or stacked *β*-sheet rich structures [32–36]. The conformational switch can be gauged through their susceptibility to proteases [37]. To determine the region of Orb2A PLD that adopts distinct conformational states, we inserted the tobacco etch virus (TEV) protease recognition motif (ENLYFQG) at the N-terminus of EGFP-tagged Orb2 protein (Fig 2A), and we measured the accessibility of the TEV protease to these sites [38]. Insertion of the TEV protease sites at the positions indicated in Fig 2A did not alter the ability of Orb2 to oligomerize (S2A and S2B Fig) or bind to mRNA (S2C Fig). We found that the TEV protease site located outside the N-terminal of Orb2A or Orb2B (Orb2A162TEV, Orb2A216TEV and Orb2B370TEV) was cleaved efficiently and equivalently in both the oligomeric and the monomeric forms (Fig 2B). However, when the TEV site was located within the first 162 residues of Orb2A (Orb2A88TEV), or within the first 242 residues of Orb2B (Orb2B242TEV), most of the oligomers and a fraction of the monomers were not cleaved by TEV after 24h incubation with TEV protease (Fig 2B). This differential cleavage of monomers implies that: i) the 88 N-terminal residues are not inherently inaccessible; and ii) there is a conformational variability even among monomeric forms.

To further explore the conformational variability in the monomer population and to rule out the possibility that resistant monomers were not simply oligomers that have dissociated, we obtained protein fractions containing primarily monomeric Orb2 by differential centrifugation. When the TEV protease site is within the first 162 residues of Orb2A, only a fraction of the monomeric protein is accessible to TEV (S3 Fig). These results suggest that the N-terminal Q/N rich region that ends at residue 162 is most likely the boundary of the PLD and that this domain can adopt at least two distinct conformational states: a protease-accessible monomeric state and a protease inaccessible state in both monomeric and oligomeric forms (Fig 2B and S3 Fig).

### Dissection of the Orb2A PLD

PLDs are often organized such that two distinct regions mediate the initiation of aggregation and recruitment to the aggregate for self-perpetuation. To determine whether the Orb2A PLD has this organization, we studied the recruitment of red-labelled proteins into EGFP-labelled preformed aggregates using a cell fusion-based assay [39]. Three distinct Orb2AΔPLD-Cherry fluorescent protein constructs that lacked specific regions of the PLD were generated: Orb2AΔ1-88-Cherry, Orb2AΔ88-162-Cherry, or Orb2AΔ1-162-Cherry. We fused S2 cells carrying Orb2A-EGFP to Orb2AΔPLD-Cherry expressing cells by inducing the expression of the *Caenorabditis elegans* epithelial fusion failure 1 (EFF-1) protein [39], which causes cell fusion via a homotypic interaction and the mixing of cytoplasmic but not nuclear components (Fig 2C). While Orb2AΔ1-88-Cherry expression appeared mostly diffuse by itself, in the fused cells this protein variant formed large puncta (Fig 2D). Orb2AΔ88-162-Cherry formed few puncta by itself but co-aggregated efficiently with full-length Orb2A-EGFP to form large fluorescence puncta. By contrast, Orb2AΔ1-162-Cherry, which lacks the entire PLD, failed to form any such aggregates (Fig 2D). Since only Orb2A-EGFP efficiently induced the formation of Orb2AΔ88-Cherry puncta but not EGFP, Orb2B-EGFP, or Orb1-EGFP, these results indicate that the intramolecular interaction is highly specific (Fig 2E). Surprisingly, the Orb2A protein from *D*. *willistoni*, which is ~81% identical to the *D*. *melanogaster* protein, forms puncta when it is expressed in S2 cells. However, Orb2A from *D*. *willistoni* failed to induce the aggregation of Orb2AΔ88-Cherry into puncta (Fig 2D), indicating that this is a species-specific process. These data suggest that the organization of Orb2A’s PLD resembles that of other prion-like proteins, whereby the first 88 residues are important for initiating aggregation, and residues 88–162 are important for the recruitment into preformed aggregates.

### A Single Point Mutation in Orb2A Affects Its Amyloidogenic and Prion-like Properties

One surprising feature of PLDs is that although the domains can substitute each other, the amino acid sequence of the various PLDs are distinct. Therefore, it remains unclear to what extent individual amino acids at various positions contribute to the prion-like properties. Intriguingly, previously we found that a F to Y substitution at the 5th position of the Orb2A PLD—representing the addition of a single hydroxyl group—dramatically reduced Orb2 oligomerization and impaired memory consolidation [11]. This prompted us to determine the role of the 5th residue in amyloid formation and what aspects of amyloid formation (e.g., recruitment) and/or prion-like behaviour (i.e., self-sustaining properties) are affected by this mutation.

**Fig 3:**
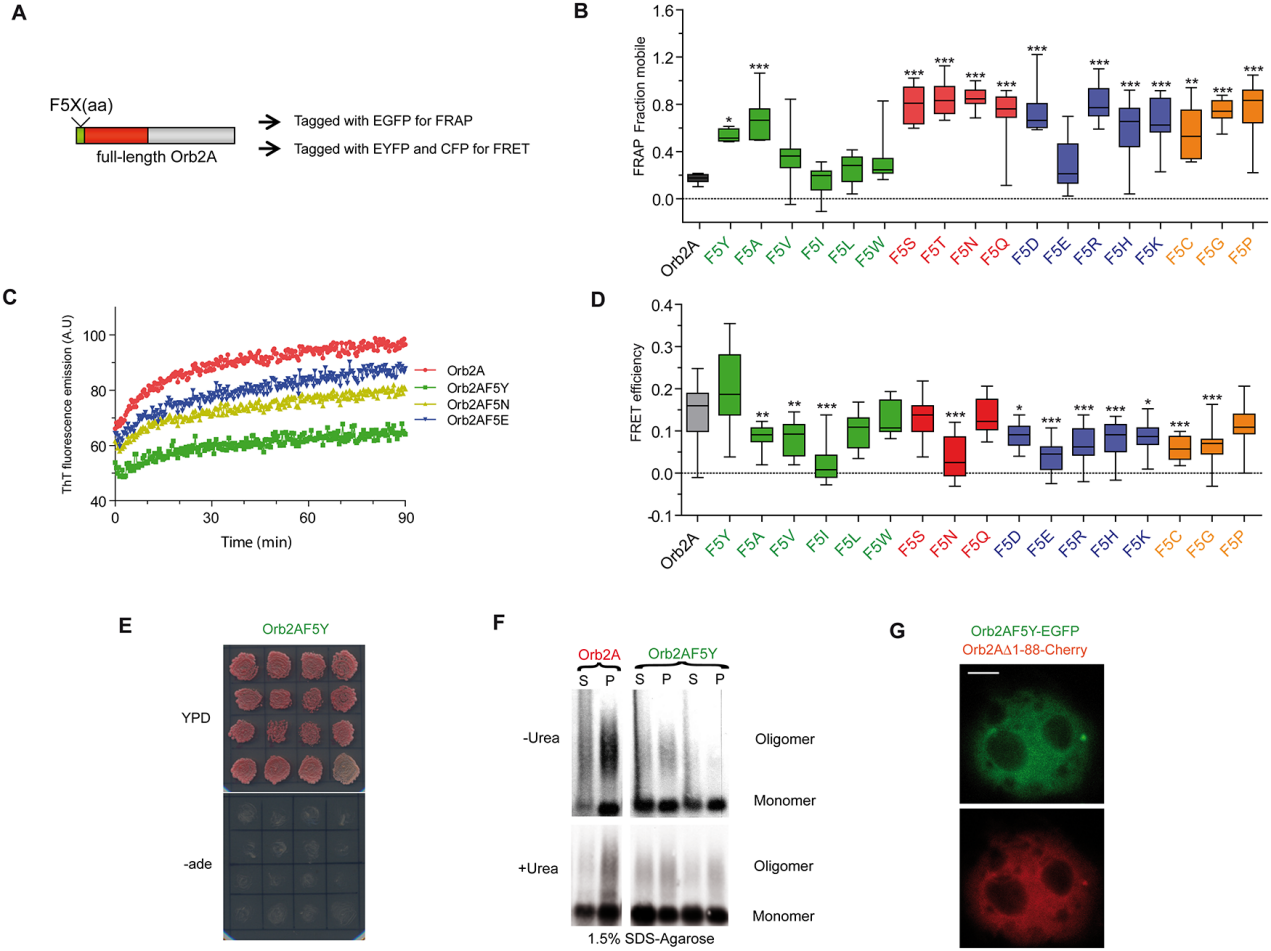
The F5 residue in Orb2A is critical for Orb2 amyloid-like oligomer formation and self-sustaining prion-like properties. **(A)** Schematic representation of the experimental design and the positions analyzed by the indicated methods. **(B)** FRAP was used to measure the dynamic nature of the Orb2A-EGFP aggregates, showing that substitution of the 5th residue makes the aggregates more dynamic. **(C)** The F5Y mutation reduces the rate of amyloid formation, as gauged by ThT fluorescence. **(D)** FRET was used to measure the relative orientation and organization of the Orb2A proteins in the aggregate. For Fig 3B and 3D analysis, data are represented as mean ± SEM. We assumed statistical significance at *p < 0.05, **p < 0.01 and ***p < 0.001 (One-way ANOVA). **(E)** A single residue substitution in Orb2A, F5Y, dramatically reduced the ability to adopt the prion-like state. **(F)** Orb2A harbouring the F5Y mutation partitions less into the pellet fraction, and it forms less SDS-resistant oligomers than the wild type form. **(G)** Orb2AF5Y-EGFP did not induce oligomerization of Orb2A lacking the 88 N-terminal residues (Orb2AΔ1-88-Cherry). Scale bar: 5 *μ*m. The underlying data for panels in this figure can be found in S1 Data.

To address these issues, the F5 residue was substituted for 18 different residues (Fig 3A), and Orb2A puncta stability was quantified using fluorescence recovery after photobleaching (FRAP). Remarkably, substitution of F5 with any residue except the highly hydrophobic ones (V, I, L, or W, only exception being E) strongly destabilized Orb2A oligomers (Fig 3B). ThT binding to Orb2A variants correlated with FRAP results, and the Orb2AF5Y mutant showed the weakest enhancement in ThT fluorescence (Fig 3C). To determine whether this 5th residue might also play an important role in oligomer packing, in addition to affecting the rate of Orb2A oligomerization, we performed fluorescence resonance energy transfer (FRET) assay. FRET efficiency depends on the distance between the fluorophores, which in turn depends on the orientation and packing of the protein subunits in the homo-oligomer. Surprisingly, only the F5L, F5W, F5S, F5Q and F5P (One-way ANOVA, *p* > 0.05) substitutions produced an average FRET efficiency similar to the efficiency observed in the wild-type protein. Moreover, the F5Y substitution produced aggregates with very variable FRET efficiency, whereas all other substitutions decreased the average FRET efficiency (*p < 0.05), suggesting that the nature of the 5th residue is important in oligomer packing (Fig 3D). Thus, the 5th residue of Orb2A seems to mediate a key intramolecular interaction important for the oligomerization process [40].

We then asked whether this perturbation in oligomer packing and stability affected the prion-like behaviour of Orb2A (see Fig 1H). Remarkably, this single residue substitution dramatically interfered with the capacity of the Orb2A’s PLD to adopt a stable prion-like state, as concluded from a chimeric yeast prion assay (Fig 3E), coinciding with the reduction in the SDS-urea resistant amyloidogenic oligomeric states (Fig 3F). The Orb2AF5Y-EGFP mutant also impaired the formation of Orb2AΔ1-88-Cherry puncta (Fig 3G), and this PLD variant exhibited impaired capability to aggregate, as measured by turbidimetry and far-UV CD spectroscopy (S4A and S4B Fig). This mutant also has decreased ability to form amyloid components, according to a CR binding assay (S4C Fig) and oligomers/fibers, as assessed by TEM (S4D Fig). Taken together, these results corroborate the critical role of the F5 residue of Orb2A for its self-sustaining recruitment and amyloidogenic properties reported to be necessary for memory consolidation [11]. These results also suggest that there are inherent structural features determined by specific amino acid residues that control the Orb2A amyloid formation.

### Orb2 Shares Common Features with the Pathological Amyloidogenic Pathway

The possible existence of intrinsic differences (related to protein structure or/and dynamics) between functional and pathological amyloidogenic pathways prompted us to carry out a comprehensive analysis on the various stages of amyloid formation.

The monomeric species of amyloidogenic proteins represent the starting point and a key determinant of the amyloidogenic cascade. The conformational changes that occur in the monomeric protein are difficult to study with bulk structural techniques because of the coexistence of many conformational states in a dynamic equilibrium. However, single-molecule techniques, like atomic force microscopy-based single molecule force spectroscopy (AFM-SMFS), allow the determination of the conformational diversity of these monomers [41]. We focused on the disordered region comprising the first 162 amino acid residues of Orb2A-PLD (S5A Fig) as the sequence that is necessary and sufficient for prion-like behaviour and amyloid formation, as well as for memory consolidation [21]. We performed AFM-SMFS in the length-clamp mode (at a constant pulling speed of 0.4 nm/ms) in order to compare the amyloidogenic properties of Orb2A PLD with those of pathological amyloids at the monomer level [41]. As a single-molecule reporter, we constructed a fusion protein whereby the Orb2A PLD was mechanically protected inside the fold of a reference protein (carrier), rendering it amenable to unequivocal single-molecule analysis (S5B Fig). Prior to AFM-SMFS analysis, to verify that both the PLD region and the carrier in the fusion protein preserve their structural integrity, we performed 1D ^1^H and 2D ^1^H nuclear Overhauser effect spectroscopy (NOESY) nuclear magnetic resonance (NMR) and far-UV CD spectroscopies (S5C–S5E Fig). Furthermore, turbidimetry analysis, CR binding, and negative-staining TEM experiments corroborate that the PLD region maintains its amyloidogenic properties when protected inside the fold of the carrier (S6 Fig).

**Fig 4:**
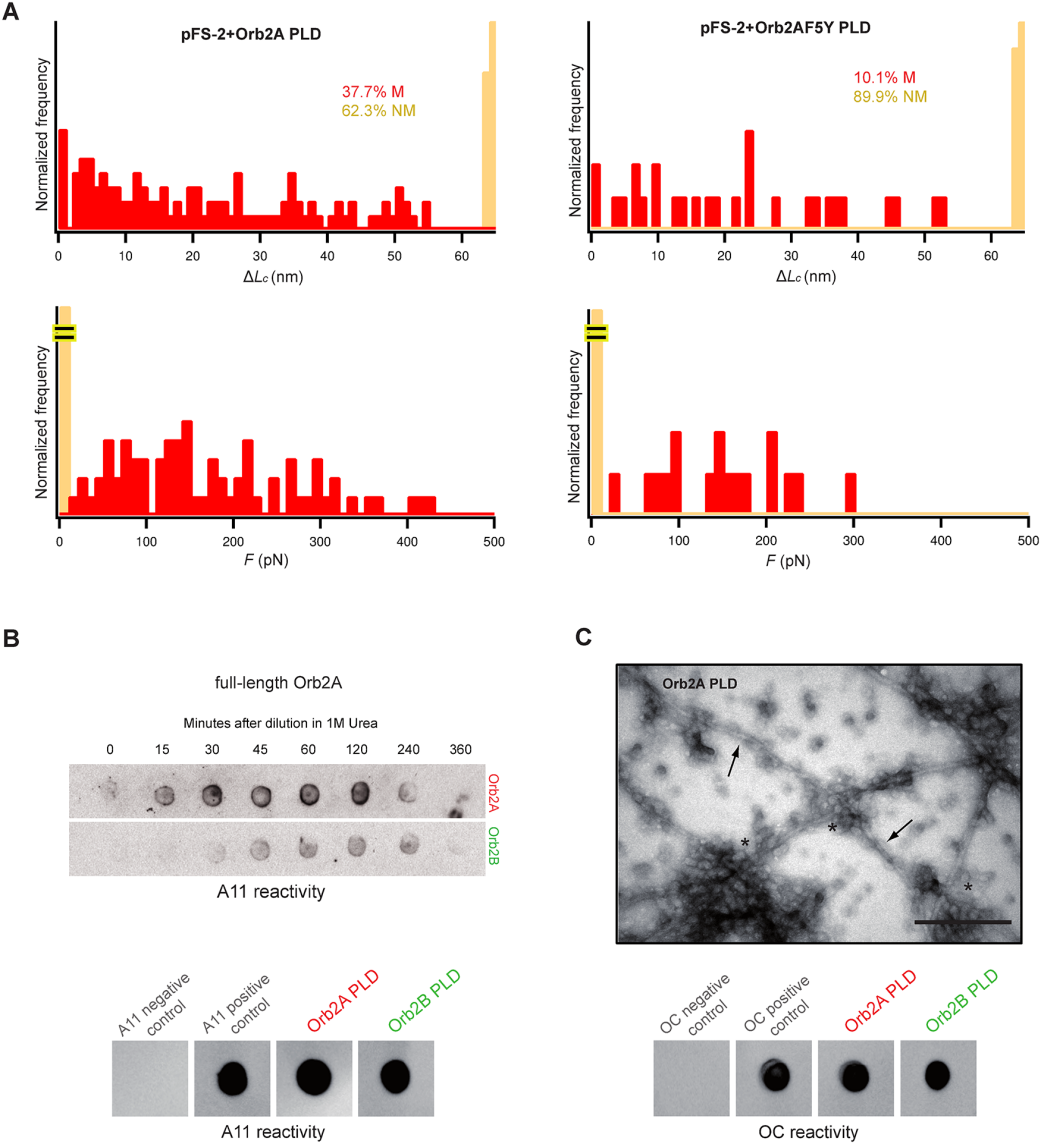
Orb2 shares common features with the pathological amyloidogenic pathway. **(A)** Δ*L*_*c*_ and *F* SMFS histograms of pFS-2 polyproteins carrying the Q/N-rich PLD of Orb2A show a broad mechanical polymorphism in terms of the increased contour length (Δ*L*_*c*_, top) and mechanical stability (bottom), ranging from NM conformers (orange bars) to different M conformers (red bars, *n* = 106), similar to that found in pathological amyloids [41]. In line with its reduced ability to form amyloids (see Fig 3 and S4 Fig), the mechanical conformational polymorphism of the F5Y mutant is diminished, increasing the proportion of NM conformers (*n* = 109). **(B)** Immunodot blot showed that like toxic oligomeric intermediates of other amyloidogenic proteins, both full-length Orb2A, and to a lesser extent Orb2B, as well as their isolated PLDs, are recognized by the A11 antibody [45]. **(C)** A representative electron micrograph of aged Orb2A PLD shows the formation oligomers (asterisks) and typical unbranched amyloid fibers (arrows) resembling those of the full-length Orb2A (see Fig 1G) and pathological amyloids. Scale bar: 0.2 *μ*m. Like pathological amyloids, those species are recognized by the fiber-specific OC monoclonal antibody [46]. For panels B and C, A*β*42 oligomers and fibers were used as positive controls for the A11 and OC antibodies, respectively, while ubiquitin was used as a negative control. The underlying data for panels in this figure can be found in S1 Data.

For SMFS analysis, after fitting the force-extension recordings to the worm-like chain (WLC) model of polymer elasticity [42,43], we found that Orb2A PLD exhibited a rich mechanical conformational polymorphism that could be classified into two general classes of conformers depending on their mechanical resistance to unfolding (*F*): nonmechanically resistant, or NM, (*F* ≤ 20 pN, 62.3% NM) and mechanically resistant, or M, (*F* > 20 pN, 37.7% M) conformers (Fig 4A, S7 Fig and S1 Table). Within the M class, a variety of monomeric conformers with different mechanical stabilities were observed. These conformers showed resistance barriers (often more than one per molecule) located at a number of positions, as measured by the length released upon unfolding (increase in contour length, Δ*L*_*c*_), indicating the high conformational diversity of Orb2A PLD (Fig 4A, S7 and S8A–S8C Fig). Like in other pathological amyloids [41], we observed extremely stable M conformers whose unfolding forces approached the values needed to break a covalent bond [44]. Intriguingly, it must be noted that some of these M conformers might be related to the prion conformers stabilized by strong noncovalent forces thought to account for prion inheritance and transmission [37]. Interestingly, the F5Y mutation showed a significantly reduced conformational polymorphism, as measured by SMFS (10.1% of M conformers; Fig 4A and S8D–S8F Fig). This reduction is in agreement with the decreased ability of the F5Y variant to form self-perpetuating amyloids (Fig 3 and S4 Fig) and the failure to stabilize memory beyond 48 h [11].

Furthermore, Orb2 shares additional features with pathological amyloids at later stages. This includes formation of toxic oligomeric species (Fig 4B) recognized by the conformational antibody A11 [45] as well as the formation of unbranched fibers recognized by the conformational antibody OC (Figs 4C and 1G), which also recognizes fibrillar oligomers and mature fibers in the A*β* assembly [46]. Taken together, these data suggest that the Orb2A PLD monomer samples a wide conformational space, consistent with our TEV protease analysis (Fig 2B and S3 Fig), and that Orb2A follows an amyloidogenic pathway reminiscent of pathological amyloids [41].

### Orb2 Forms Transient Toxic Oligomers

**Fig 5:**
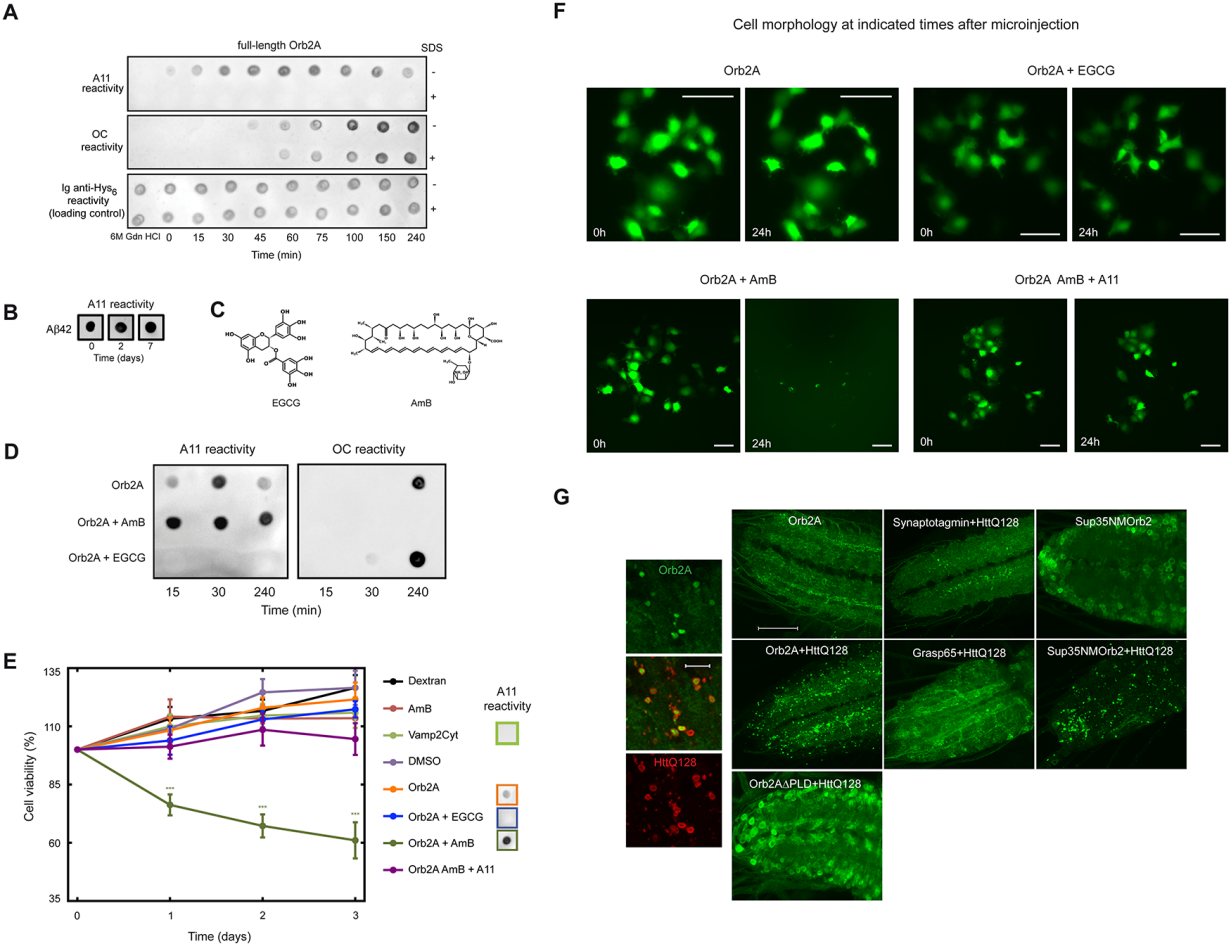
Orb2 cytotoxic oligomeric species rapidly evolve into mature amyloids and they can be kinetically trapped as well as sequestered by pathological amyloids. **(A)** Structural features during Orb2A assembly probed with conformational antibodies showed the formation of species reactive to A11 and OC antibodies. OC can exist in a SDS-resistant form, while A11-reactive oligomers are SDS-sensitive. **(B)** Lifespan of A*β*42 A11-reactive species. **(C)** Chemical structure of EGCG (left) and amphotericin B (AmB, right), used to trap OC- and A11-reactive oligomers, respectively. **(D)** Immunodot blot analysis of Orb2A:EGCG and Orb2A:AmB complexes probed with A11 and OC conformational antibodies show that oligomers trapped in the presence of EGCG interact with the OC antibody (and not with A11), whereas those formed in the presence of AmB react strongly with the A11 antibody (and not with OC antibody). **(E)** Survival curves of COS-7 cells microinjected with several samples (the A11-reactivity of which is indicated). Data are represented as the mean ± SEM: ***p < 0.001 (green asterisks, Orb2A:AmB versus remaining samples; Two-way ANOVA and Bonferroni post-test). Dextran, AmB, the intrinsically disordered region of Vamp2 (Vamp2Cyt) and dimethylsulfoxide (DMSO) are used as controls. The number of single-cells microinjected per sample was *n* = 100–200. **(F)** Fluorescence micrographs of COS-7 cells microinjected with different samples and fluorescein-labelled dextran (at 0 and 24 h after microinjection). Microinjection of the Orb2A:AmB complex resulted in a marked drop in the number of live cells at 24 h compared to those at 0 h. Notably, the A11 antibody rescued cells from apoptosis. Scale bars: 100 *μ*m. **(G)** Huntingtin protein-containing expanded polyQ repeats (HttQ128) enhances Orb2A aggregation, and this enhancement requires the Orb2A PLD. Orb2A and HttQ128 coaggregates. HttQ does not induce aggregation of other EGFP-tagged neuronal proteins. Substitution of Orb2A PLD with the NM region of yeast Sup35 showed similar HttQ-dependent aggregation. Scale bar: large panel 20 *μ*m, inset 10 *μ*m. The underlying data for panels in this figure can be found in S1 Data.

The similarities in the amyloidogenic cascade between Orb2A and pathological amyloids led us to wonder why, despite these common features, Orb2A is functional rather than toxic to the cell. One possibility is that the lifetime of the toxic oligomers formed by Orb2A and pathological amyloids is different. To test this possibility, we first used the two aforementioned conformational specific antibodies raised against A*β*: A11 [45] and OC [46]. We found that Orb2 initially formed A11-reactive SDS-sensitive oligomers that rapidly (minutes time scale) evolved to form at least two different OC-reactive species: a SDS-sensitive species and a more mature, SDS-resistant species (Fig 5A). By contrast, A11-reactive species formed by A*β*42 were stable in solution for days (Fig 5B).

In the light of these data, and to determine whether the transient Orb2 oligomers are indeed cytotoxic, we used conformational trapping with two small inhibitors (Fig 5C): EGCG [24,47] and Amphotericin B (AmB) [48]. We found that AmB slowed down Orb2A assembly and arrested it in the A11-reactive conformation, while EGCG seemed to direct the protein to the fibril state through a pathway that does not involve the formation of toxic A11-reactive oligomers (Fig 5D). To test cell viability, the different samples were microinjected into cultured cells [49]: Orb2A and the Orb2A:EGCG complex, in which Orb2A is stabilized in the OC-reactive conformation, were not cytotoxic, similar to the negative controls (Fig 5E and 5F, S9 Fig). By contrast, the Orb2A:AmB complex, which is stabilized in the A11-reactive oligomeric state, caused extensive and acute cell death 24 h after microinjection (Fig 5E and 5F, S9 Fig). Notably, incubation of the Orb2A:AmB complex with a very low concentration of the conformational A11 antibody (complex:A11, 100:1) attenuated this toxicity, indicating that toxicity was indeed due to the A11 specific conformation acquired by Orb2A (Fig 5E and 5F, S9 Fig).

To determine whether these differences in kinetic parameters are inherent features of nontoxic and toxic amyloids, we performed a domain swapping analysis between Orb2A and a disease-associated version of Huntingtin Htt exon 1 (ex1HttQ72). We compared ex1HttQ72 with two chimeric proteins in which the PLD and the Q-rich region have been swapped (S10A Fig). When the Q-rich region of ex1Htt was substituted with Orb2A PLD, it formed nontoxic, short-lived species reactive to the A11 antibody, similar to Orb2A (see Fig 5A). Conversely, the substitution of the PLD of Orb2A by the Q-rich region of the expanded ex1Htt resulted in the formation of stable A11-reactive species that exhibited a high cytotoxicity, similar to that found for ex1HttQ72 (S10B–S10D Fig). Taken together, these results suggest that Orb2A can form a toxic conformer, which structurally resembles that of toxic amyloids. However, intrinsic structural features in Orb2A-PLD renders this toxic conformation rare and transient; furthermore, it appears that Orb2A may have evolved to form mature amyloidogenic oligomers much more efficiently than pathological amyloids.

### Orb2 Can Be Trapped by Pathological Amyloids

Our observations suggest that in solution, Orb2 and polypeptides that form pathological amyloids sample similar conformational ensembles. We wondered whether in spite of being transient and rare, because of their structural similarity, these Orb2 transient toxic conformations could be hijacked by similar, yet longer-lived conformers from neurotoxic amyloids. To test this hypothesis, we coexpressed EGFP-tagged Orb2A protein with the Huntingtin protein-containing expanded polyQ repeats (HttQ128) in the larval motor neuron. HttQ128, unlike Orb2A, is neurotoxic, and its chronic expression produces lethality [50]. In contrast to when it is expressed alone, Orb2A formed larger fluorescence puncta in the presence of HttQ128, indicative of its aggregation, and in some cases these Orb2A puncta were surrounded by HttQ128 protein (Fig 5G and S11A Fig). The effect of HttQ128 on Orb2 appears to be specific, given that it had no obvious effect on other EGFP-tagged proteins, such as the synaptic protein synaptotagmin or the Golgi-associated protein GRASP-65, used as negative controls (Fig 5G and S11B Fig). Similarly, removal of the PLD from Orb2A abrogated its HttQ128-enhanced aggregation (Fig 5G). Since the structural properties of Orb2A are similar to those of the NM PLD of yeast prion Sup35, we also studied a chimeric protein in which the Orb2A PLD was substituted with the Sup35 PLD. Consistent with the structural studies, this chimeric construct was also recruited into the HttQ128 aggregates (Fig 5G and S11C Fig). These results suggest that pathological amyloids, when present in excess, can nucleate Orb2A aggregation, presumably by capturing the transient toxic conformers of Orb2 proteins.

### A Small Anti-amyloidogenic Peptide Blocks Orb2 Amyloidogenesis

Polyglutamine-binding peptide 1 (QBP1) is a known inhibitor of the amyloidogenesis of HttQ expansions. This hydrophobic peptide binds monomeric expanded polyQ proteins and blocks the initial critical *β*-conformational transition of these species, suppressing their subsequent oligomerization and fibrillogenesis, and consequently, the associated cytotoxicity and neurodegeneration [41,49,51–53]. QBP1 acts as a polyvalent anti-amyloidogenic agent on several amyloids [41], and thus, we examined the potential inhibitory effect of the minimal active core of QBP1 (Ac-WKWWPGIF-NH_2_) [54] on Orb2A amyloid formation. The conformational similarity at the monomer level between Orb2 and HttQ prompted us to further examine whether QBP1 could inhibit Orb2A amyloid formation.

**Fig 6:**
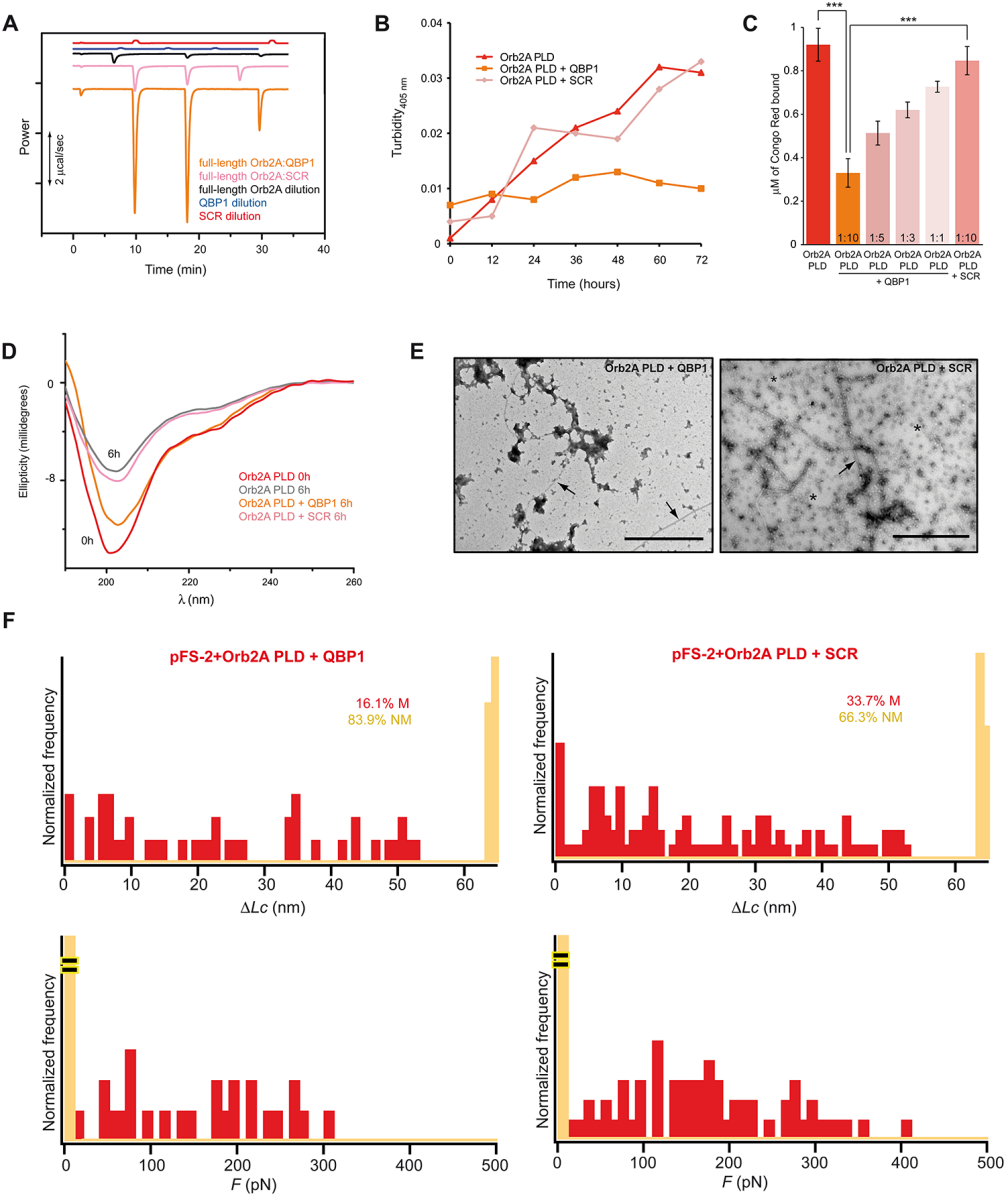
QBP1 interferes with Orb2 amyloid formation in vitro. **(A)** Representative calorimetric traces for the interaction of full-length Orb2A with the QBP1 and SCR peptides. Traces in orange and pink correspond to the heat released upon injection of Orb2A into the ITC cell loaded with an excess of QBP1 or SCR, respectively, while black, blue, and red traces correspond to the Orb2A, QBP1, and SCR dilutions, respectively. **(B)** QBP1 but not SCR drastically reduced the turbidity at 405 nm of aged Orb2A PLD. **(C)** QBP1 reduces the quantity of amyloid formed as shown by measuring the CR bound to Orb2A PLD. The data are represented as the mean ± SEM: *p < 0.05 and ***p < 0.001 (One-way ANOVA and Tukey post-test). **(D)** Far-UV CD spectroscopy in the absence or presence of QBP1 or SCR indicates that QBP1 blocks protein precipitation over time of Orb2A PLD, but SCR does not. **(E)** Representative electron micrographs show that oligomers (asterisks) and amyloid fibers (arrows) of Orb2A PLD were drastically reduced when incubated with QBP1 (left) but not with the SCR (right). Scale bars: 1 *μ*m. **(F)** The conformational polymorphism of Orb2A PLD is strongly diminished in the presence of a 1:10 molar excess of QBP1, which decreases the M frequency relative to the NM conformers (QBP1, *n* = 112 and SCR, *n* = 101). The underlying data for panels in this figure can be found in S1 Data.

Isothermal titration calorimetry (ITC) revealed that QBP1 physically interacts with Orb2A, and that complex formation, a slow event, was exothermic in nature (Fig 6A). Under similar conditions, a scrambled version of QBP1 (SCR, Ac-WPIWKGWF-NH_2_) [54] interacted poorly, serving as a negative control in the subsequent experiments (Fig 6A). Unlike the SCR, incubation with QBP1 inhibited Orb2A PLD aggregation and amyloidogenesis, as monitored by turbidimetry and CR binding (Fig 6B and 6C). Far-UV CD spectroscopy revealed that QBP1, but not SCR, suppressed Orb2A PLD signal loss due to protein precipitation (Fig 6D). Furthermore, TEM showed a significant reduction in the formation of both oligomers and fibers in the presence of QBP1 but not the SCR (Fig 6E) compared to Orb2A PLD alone (see Fig 4C). Consistent with these results, the SMFS analysis revealed that Orb2A PLD treated with QBP1 formed fewer M conformers (from 37.7% to 16.1%), shifting the NM/M proportion towards an increased population of NM conformers, while SCR had no effect on this initial conformational transition (Fig 6F and S12 Fig). Together, these results indicate that QBP1 interferes with Orb2 amyloid formation in vitro and suggest that early in amyloidogenesis, Orb2 and some pathological amyloid-forming polypeptides adopt similar conformations that are recognized and blocked by QBP1.

### QBP1 Interferes with Memory Consolidation

**Fig 7:**
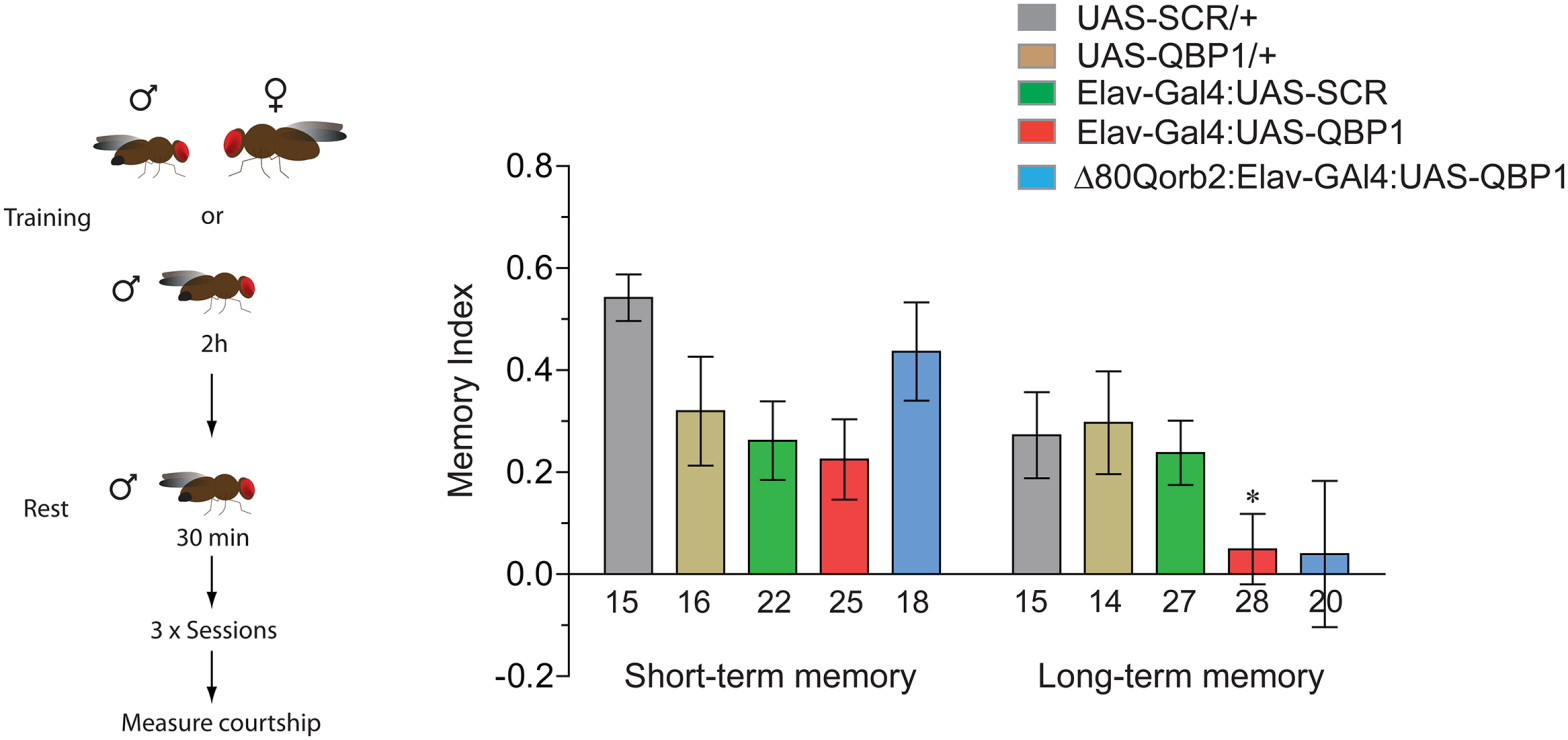
QBP1 interferes with Orb2-mediated memory consolidation in vivo. Pan-neuronal expression of QBP1 disrupts long-term male courtship suppression memory, while it does not interfere with short-term memory. Expression of QBP1 in the Δ80Q orb2 mutant background did not have any additive effect on long-term memory. The data are represented as the mean ± SEM. We assumed statistical significance at *p < 0.05 (One-way ANOVA). *n* = 16, 15, 25, 22, 18, 14, 15, 28, 27, 20 from left to right. The underlying data for panels in this figure can be found in S1 Data.

Based on the fact that QBP1 inhibits the transition from the monomeric Orb2A state to conformations that lead to amyloid formation, we tested in *D*. *melanogaster* the physiological consequences of QBP1 expression on memory consolidation. To this end, we used the male courtship suppression memory paradigm, in which a virgin male repeatedly exposed to unreceptive females learns to suppress courtship towards them, and this learned suppression persisted for days. Using the Gal4-UAS system, the QBP1-cyan fluorescent protein (CFP) or SCR-CFP peptides were expressed panneuronally [53]. QBP1 expression in the nervous system, or that of the control SCR, did not affect fly development, although normal courtship behaviour was slightly dampened (S13 Fig). Following training, both experimental flies (Elav-Gal4:UAS-QBP1-CFP or SCR-CFP) and the genetic controls (Elav-Gal4 and UAS-QBP1 or UAS-SCR) displayed a similar suppression of courtship immediately after training, suggesting that the expression of these peptides does not interfere with learning or short-term memory. However, when measured after 24 h, the Elav-Gal4:UAS-QBP1 males displayed elevated courtship compared to the Elav-Gal4:UAS-SCR control flies, indicative of a loss of long-term memory (Fig 7). To determine whether QBP1-mediated memory loss is independent of Orb2, we expressed QBP1 in Δ80QOrb2 flies that lack N-terminal Q-rich 80 amino acid residues of Orb2 (Fig 7). The Δ80QOrb2 flies form short-term memory but no long-term memory [11,17]. Expression of QBP1 in Δ80QOrb2 had no additive effect in the loss of long-term memory (Fig 7). Since QBP1 is a low affinity peptide that can only block early stages of oligomerization but not already formed oligomer, we also investigated the consequence of transient expression of QBP1 in courtship suppression memory. To this end, we used RU486-inducible GeneSwitch Elav-Gal4 system and induced expression of the QBP1 peptide in the adult flies 24 h before training [55]. Transient expression of QBP1 had no effect on the long-term courtship suppression memory (S14 Fig). Finally, to determine whether chronic expression of QBP1 results in general disruption of the nervous system, we trained the ElavGal4-UASQBP1 flies in a heat-box paradigm (S15 Fig). In this operant conditioning paradigm, flies learn to avoid one side of an otherwise symmetrical chamber [56]. Intriguingly, the heat box paradigm produces robust short-term memory, but the memory does not persist beyond an hour or two. The memory curve of QBP1 was similar to that of wild type flies, suggesting that QBP1 expression does not interfere with the animal’s ability to form short-lived memories (S15 Fig). Taken together, these results suggest that chronic QBP1 expression can interfere with some form of long-term memory and the effects of QBP1 in memory may be partly mediated through Orb2.

## Discussion

Amyloids were discovered and primarily studied in the context of disease or nonfunctional states of proteins. The unique structural features of amyloids set them apart, endowing them with extraordinary stability and, in some cases, the unique capacity to self-perpetuate in a dominant manner [37]. The recent discovery of functional amyloids raises some fundamental questions as to what makes some amyloids pathological and others beneficial, how similar the structures of pathological and functional amyloids are, and how they are formed.

### What Separates Toxic and Nontoxic Amyloids?

**Fig 8:**
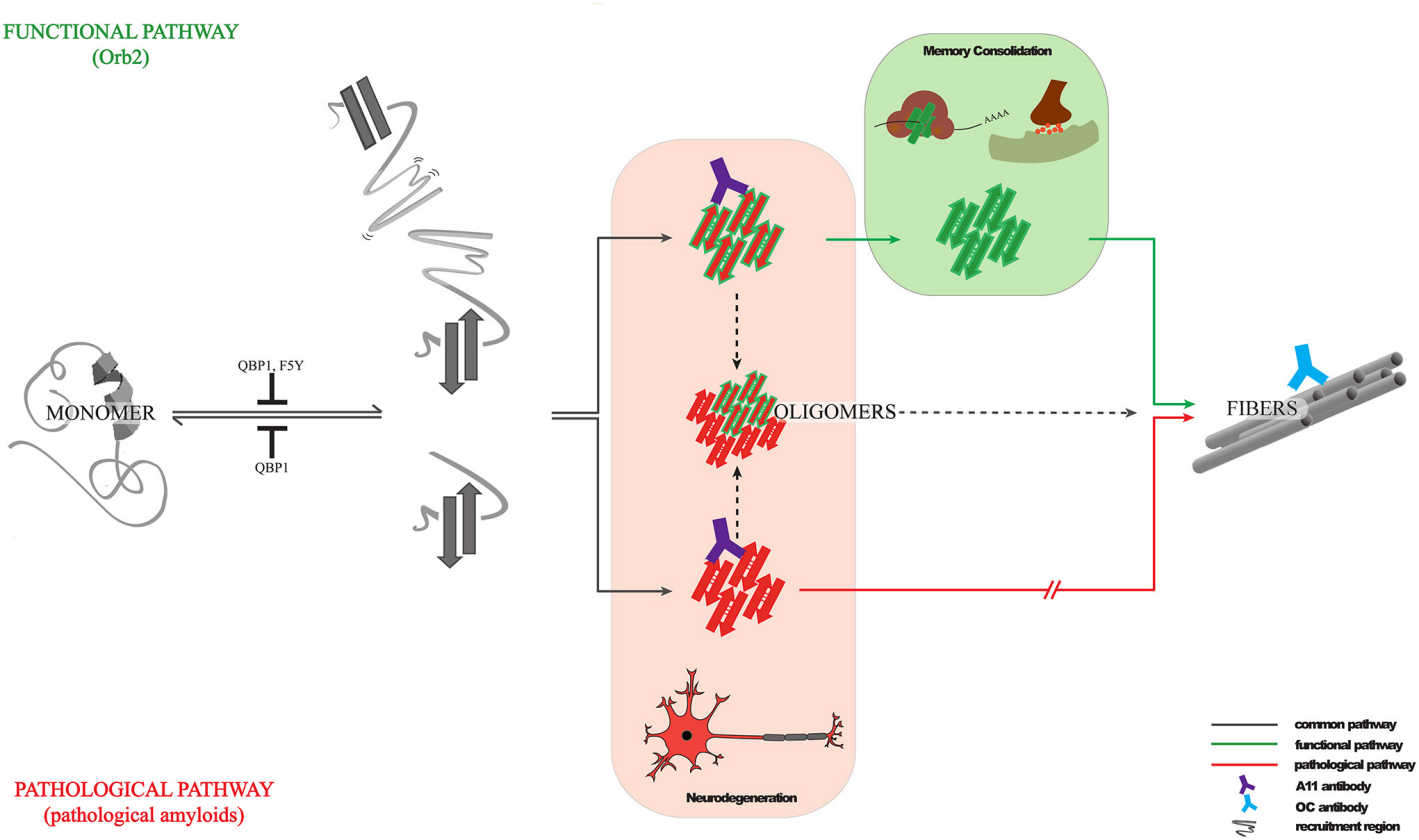
Structural basis of memory consolidation mediated by Orb2 amyloid. In *Drosophila*, the Orb2 protein (**top**) is a functional amyloid with self-sustaining prion-like properties that follows an amyloidogenic pathway resembling that of pathological amyloids (**bottom**). During the assembly pathway, Orb2 and pathological amyloids can form A11-reactive toxic oligomers, and Orb2 can be sequestered by pathological amyloids to form hetero-oligomers. However, intrinsic structural features make Orb2 toxic oligomers rare and transient, suggesting that this functional amyloid appears to have been honed by evolution in adopting a self-sustaining amyloid-like state much more efficiently than pathological amyloids in order to avoid cytotoxicity and perform its role in memory consolidation. This function is supported by the amyloid state of Orb2, since its inhibition by an anti-amyloidogenic peptide (QBP1) selectively interferes with the long-term persistence of memory.

Here we have analyzed the structural states of Orb2A, an amyloidogenic protein that is important for memory consolidation. We find that Orb2A not only forms self-perpetuating amyloids but it does so extremely quickly and efficiently. Surprisingly, Orb2A has several structural features and properties that are similar to those of pathological amyloids (Fig 8). First, the Orb2A monomer shows a rich conformational polymorphism with mechanical stabilities similar to those of the well-characterized pathological amyloids [41]. Second, Orb2 binds to the conformational antibody A11, which detects the toxic oligomeric species of pathological amyloids [45]. Third, like pathological amyloids, Orb2A can form unbranched amyloid fibers and pre-fibrillar oligomers that react against the conformational antibody OC [46,57–60]. Finally, Orb2A amyloid formation is inhibited by QBP1, a peptide capable of inhibiting amyloid formation in expanded polyQ tracts [41,51].

What makes Orb2A PLD beneficial despite sharing all these features with pathological amyloids? Orb2 amyloid-like oligomers are formed in response to neuronal stimulation to support memory, which indicates the existence of a highly regulated mechanism in the cell to drive Orb2 amyloid formation and most likely to avoid the toxicity of its A11-reactive oligomers. One such regulation is the tight control of the Orb2A protein level via phosphorylation [20] and possibly other mechanisms. Here we show the existence of additional intrinsic kinetic factors in Orb2A, i.e., the lifespan of A11-reactive oligomers, which is probably related to its efficiency to form a functional amyloid state. We postulate that although the early events in Orb2 amyloid formation are similar to those of pathological amyloids, in subsequent steps it acquires structural features to rapidly evolve to a nontoxic amyloid-like state (Fig 8). For Orb2 proteostasis, these intrinsic structural features could be further enhanced in the cell by association with other components or by the direct modification of proteins, such as phosphorylation [20]. Both the presence of A11-reactive oligomers and the ability to form amyloids efficiently have been also reported for the intracellular nonpathological amyloids of the yeast Sup35 prion [48], which suggests that this may be a general mechanism for some amyloids to avoid cytotoxicity. Still, it must be noted that neuronal CPEB/Orb2 is the only known functional amyloid-like protein in the nervous system. This distinction is fundamental for two reasons: first, the nervous system is particularly susceptible to amyloid-based disease and; second, amyloids are known to interfere with cognitive abilities including memory.

### Amyloid-like States as a Stable Substrate of Memory

The persistence of memories over months and years raises the fundamental question of how memory is protected against molecular turnover [61]. Structural changes in individual proteins and in supramolecular protein assemblies have been proposed to play an important role in long-term memory [62,63]. The prion features of the specific neuronal CPEB isoform, such as Orb2 in *Drosophila* [11], or CPEB3 in mouse [9,12] that can adopt a self-sustaining state at synapses very rapidly, provide a plausible solution as to how memories can remain stable in the face of constant molecular turnover [10,16,64]. Although exactly to what extent the self-sustaining amyloidogenic properties observed in vitro reflect the in vivo properties remains unclear, accumulating evidence reinforces the hypothesis that an amyloid-like state of neuronal CPEB is involved in long-lasting memory. In this regard, the present study provides evidence that mutations in Orb2, such as the 5th residue in Orb2A, that are known to interfere with memory consolidation also impair the protein’s ability to attain a self-sustaining amyloidogenic state as monitored by in vitro measurements. Similarly, the expression of QBP1, an amyloid-blocking peptide, impairs memory consolidation (Fig 8). However, we are not sure whether the memory-disrupting effect of QBP1 is applicable for all forms of long-term memory or whether the effects of QBP1 in memory are mediated exclusively through the Orb2 protein. Furthermore, in the absence of atomic-level 3D structural analysis of endogenous CPEB protein in the amyloid-like state, it remains unclear exactly to what extent they are similar to toxic amyloids.

Our observations also highlight certain features of functional amyloids that may be relevant for neuropathologies of amyloid-based diseases. First, the short life span of Orb2 toxic oligomers supports a therapeutic approximation based on speeding up the conversion of toxic oligomers into stable amyloids [65]. Second, Orb2’s capacity to form toxic conformers resembling those of pathological amyloids, and its sequestration into HttQ128 aggregates (Fig 8), suggests that pathological amyloids might hijack Orb2 into a nonfunctional state, impairing its physiological activity. Third, anti-amyloidogenic compounds that target early stages of amyloidogenic pathways might inadvertently interfere with functional amyloid formation, impairing their associated function. Finally, the polyvalent amyloid blocker QBP1, or improved analogues based on its recently determined structure [66], could be used in the future to block newly formed traumatic memories (Fig 8) [67]. However, we are cognisant of the inherent limitations of our ideas since none of our studies are carried out with human proteins in the human nervous system. These anticipated connections of our in vitro findings with memory need to be explored in detail.

## Materials and Methods

### ThT Binding Assay

The ThT binding assay was performed essentially as described previously [68]. Briefly, 400 picomoles of protein/100 *μ*l aliquot were taken at the time indicated and mixed with 700 *μ*l of 25 *μ*M ThT (Sigma) in 50 mM Glycine buffer (pH 8.5). The reaction mix was excited at 442 nm, and the emission at 482 nm was measured using a Fluoromax-4 spectrofluorimeter, and three independent measurements were taken for each sample. To obtain the ThT enhancement fluorescence due to Orb2 alone, the 6 M GdnHCl-PBS buffer was dialyzed similarly, and for each time point, the fluorescence of ThT in this buffer was subtracted.

### FTIR Spectroscopy

FTIR spectroscopy was carried out using the same His-tagged recombinant proteins used in ThT binding assay. FTIR was performed with a Nicolet 6700 spectrometer equipped with a ZnSe multibounce ATR accessory. The instrument was corrected for ATR penetration. Spectra were collected at 2 cm^-1^ resolution and averaged over 64 scans. Samples were precipitated by addition of methanol to a final concentration of 50% and incubated overnight at 4°C. Pellets were spread on the ATR and dried with a stream of air before each measurement. Spectra were corrected for water vapor by subtracting the background collected immediately after each scan scaled to fit the local shape of the spectrum in the 1,600–1,700 cm^-1^ region. The liquid water spectrum was subtracted by matching the signal at 2,200 cm^-1^ to a liquid water spectrum via linear least squares. A linear background between 1,580 and 1,720 cm^-1^ was subtracted, and the spectrum in this region was normalized to obtain the final spectrum. The resulting spectra were fit to four Gauasian peaks with nonlinear least squares. All subtractions and fittings were performed using custom plugins written in ImageJ (NIH, Bethesda, MD) and are available at http://research.stowers.org/imagejplugins. The 1,620 cm^-1^ band with a width of ~13 cm^-1^ was assigned to extended cross-*β* sheet structures only found in amyloid proteins. The 1,637 cm^-1^ band with a width fixed at 13 cm^-1^ was assigned to typical *β*-sheet structures such as those found in concanavalin-A. The 1,655 cm^-1^ band with a width fixed at 13 cm^-1^ was assigned to random coil and *α*-helical structure. Finally, the 1,678 cm^-1^ band with a width fixed at 13 cm^-1^ was assigned to turns. In addition, FTIR was performed after incubation of the precipitated pellets in D_2_O for 1 h, and a minimal change in the *α*-helical content was observed, suggesting the absence of pervasive random coil structure.

### Self-Sustaining Yeast Prion Assay

For the chimeric yeast prion assay, we have used the methods developed previously [69]. Briefly, the N-terminal domains of Orb2A, Orb2B, and Orb1 were optimized for expression in yeast and cloned into TopoDonor vector (Invitrogen). Using pAG414SUP35-ccdB-SUP35C (LEU, CEN plasmid, Sup35 promoter, SUP35C domain) Gateway vector and LR clonase (Invitrogen), the N-terminal domains, were fused in frame to Sup35C domain to create the chimeric construct [69]. The Orb2-Sup35C (LEU selectable marker) construct was introduced into W303aΔsup35 strain [MATa; leu2-3, 112; his3-11,-15; trp1-1; ura3-1; ade1-4; can1-100; SUP35::HygB; [*psi*-];[*PIN*+]] via plasmid shuffling. The yeast were grown in YPD media and plated on either YPD-agar or SC-agar lacking adenine and the [*PSI*+] colonies were selected 2–3 d after plating. Selected colonies were grown in YP-glycerol plates to avoid petites. To discard that phenotypes are not due to mutation in the Orb2-Sup35C, the plasmid was isolated from both cell types and sequenced, and white phenotypes were rescued by transforming the cells with plasmid expressing just the C-terminal domain of Sup35 protein. To determine the heritability of the prion-like state, individual red or white colonies were streaked for multiple times.

### Differential Centrifugation of the Cell Extract and the TEV Protease Assay

S2 cells expressing the Orb2A-EGFP, Orb2B-EGFP, Orb2A88TEV-EGFP, Orb2AA216TEV-EGFP, Orb2B242TEV-EGFP, or Orb2B370TEV-EGFP constructs were lysed in PBS buffer containing 0.1% NP-40 and protease inhibitors (Roche). The lysate was centrifuged at 10,000 × g for 15 min to remove the cell debris and nuclei, and the supernatant was then centrifuged at 174,000 × g in a TLA120.2 rotor for 2 h. The supernatant was collected, and the pellet fraction was resuspended in PBS + 0.1% NP40 buffer in a volume equal to the supernatant. For western blotting, 7.5 *μ*l of the lysate, and 15 *μ*l of the supernatant and pellet fractions, were analyzed by SDS-PAGE. For TEV protease digestion, approximately four times the volume of the pellet fraction was used compared to the supernatant fraction to normalize for the amount of Orb2A and Orb2B present. TEV digestion was performed overnight at 4°C with 1 *μ*g of recombinant purified TEV protease. To analyze Orb2A-EGFP and Orb2B-EGFP from fly heads, the ultracentrifugation protocol was modified by using 1% Triton X-100 lysis buffer instead of the NP-40/PBS buffer.

### Antibodies

All anti-Orb2 antibodies were raised against recombinant six histidine-tagged full-length Orb2A proteins. The antibody 273 was raised in rabbits (Covance), and antibody 2233 was raised in guinea pigs (Pocono). All antibodies were affinity purified against Orb2A protein. For western blots, the antibodies were used at the following dilutions: Ab273, 1:1,000 dilution; Ab2233, 1:2,000 dilution; and Ab402, 1:2,000 dilution. The chicken anti-EGFP antibody (abCAM) was used at 1:5,000, and all secondary antibodies (Cell Signalling) were diluted 1:10,000.

### Western Blotting and Immunoprecipitation

For all biochemical analyses, freshly prepared head extracts were used unless mentioned otherwise. For western blotting, fly heads were homogenized (2–4 *μ*l of buffer/head) in PBS buffer I, containing: 150 mM NaCl, 3 mM MgCl_2_, 0.1 mM CaCl_2_, 5% glycerol, 1% Triton X-100, and protease inhibitors (Roche). Where applicable, the extracts were also treated with 50 ng/ml of purified RNaseA (Qiagen). The total homogenate was incubated at 4°C for 30 min with rotation, centrifuged at 10,000 × g for 10 min, and the cleared supernatant was collected. To detect Orb2 protein, extracts from approximately five head equivalents were resolved on a 4%–12% gradient SDS-PAGE (Invitrogen) or on 8% SDS-PAGE and electroblotted onto a PVDF membrane for 16 to 18 h at 30 mV in a cold room. The membranes were blocked with 5% nonfat dry milk in Tris-buffered saline (TBS)-Tween-20 buffer and incubated with the affinity-purified antibodies indicated for 12 to 16 h at 4°C with constant agitation. The antibody-antigen interaction was visualized by chemiluminescence using HRP-coupled anti-rabbit (for Ab273 or anti-guinea pig (Ab2233) secondary antibodies (Pierce).

For immunoprecipitation, fly heads were homogenized in PBS buffer I, and the lysates were clarified twice by centrifugation at 12,000 × g for 10 min at 4°C. Then, ~ 2 mg of total protein from unstimulated brain extracts and ~ 0.5 mg of total protein from stimulated brain extracts were incubated with 0.5 to 1 *μ*g of the purified antibodies for 2 h at 4°C. After 2 h, the lysates were incubated with protein A beads (Repligen) for a further 2 h, and the beads were washed five times with the PBS buffer I. The protein bound to the beads was analyzed in western blots (as described above).

### RNA Pull-Down Assay

The head extracts were prepared in a buffer containing: 20 mM Tris-HCl, (pH 8.0), 150 mM NaCl, 1 mM EDTA, 5% glycerol, 0.1% Triton X-100, 1.5 mM DTT, 0.2 mg/ml heparin, 0.2 mg/ml tRNA, 0.25% BSA, complete mini protein inhibitor (Roche), 40 units/ml RNase plus inhibitor (Promega). The RNA and protein (~2 mg of total protein) were incubated in the buffer with rotation for 40 min at RT. Pre-equilibrated Streptavidin Paramagnetic Beads (Roche) were added to each binding reaction, and the mixture was incubated for a further 40 min. The beads were then captured with a magnet, washed five times for 10 min with extraction buffer, and boiled in Laemmli SDS-PAGE sample buffer. The supernatant and pellet fractions were prepared as described above. To obtain biotin-labelled RNA, the 3’ UTR of the Oscar, Tequila, Murashka, and Actin88F genes were cloned into the TopoII vector (Invitrogen), and biotin-labelled RNA was prepared using Megascript RNA synthesis kit (Ambion) in the presence of Bio-17-ATP and Bio-11-CTP. The RNA was purified, and ~ 2 *μ*g of biotin-labelled RNA was incubated with the supernatant and pellet protein extracts. The protein/RNA complexes were recovered with streptavidin magnetic beads (Dynal). The amount of supernatant and lysate used in the control lanes is 5% the amount used for RNA binding.

### S2 Cell Transfection and EFF-1 Cell Fusion Assay

S2 cells were transfected with the constructs indicated using Effectene (Qiagen) according to the manufacturer’s instructions. The cotransfected cells were plated the next day on concanavalin-A-coated glass-bottom dishes (Mattek) and analysed by confocal microscopy. For EFF-1 fusion experiments, we cloned the ORF of *C*. *elegans eff-1* gene into the copper inducible vector pMTV5HisB (Invitrogen). The Orb2 constructs were cloned into the Pac5.1HisB vector (Invitrogen) under the constitutively active actin promoter. Cells were cotransfected with pMT EFF1 and pAc5.1EGFP-tagged ORF’s or pMT EFF-1 and pAc5 Orb2AΔ1-88-Cherry as indicated. One day later, the cotransfected cells were mixed, and fusion was initiated by adding CuSO_4_ to the media, thereby inducing EFF-1 expression. After 16 to 18 h, the cells were plated on concanavalin-A (Sigma)-coated mattek glass-bottom dishes, and the cells containing both GFP and Cherry were detected manually by confocal microscopy.

### Computational Predictions

Order/disorder prediction for Orb2A protein was made using a consensus artificial neural network prediction method, Predictor Of Naturally Disordered Regions PONDR-FIT (Molecular Kinetics). This metapredictor was developed by combining the outputs of several individual disorder predictors (PONDR-VLXT, PONDR-VSL2, PONDR-VL3, FoldIndex, IUPred, and TopIDP) and significantly improved the prediction accuracy when compared to its individual component predictors [70]. Orb2A PLD sequence was also analyzed by applying the Rosetta-Profile algorithm, which identifies sequence segments that form the steric-zipper spines of amyloid fibrils [71].

### Dot Blot Assay

Orb2 PLD samples and Orb2A/ex1Htt chimeric proteins (20 *μ*M) were incubated at 37°C without stirring in PBS (pH 7.0), and 2 *μ*l of each sample obtained at selected intervals was spotted onto a nitrocellulose membrane. The Orb2A/ex1Htt chimeric proteins were constructed by overlapping PCR cloning, using as template a clone that contains the full sequence for HttQ72 [72]. For EGCG and AmB immunodot blot analysis, the Orb2A monomer was prepared by protein denaturation in 6M GdmCl (as 0 min reference time) and then dialyzing in 5 mM potassium phosphate (pH 7.4) + 150 mM NaCl to a final concentration of 2.5 to 5 *μ*M. DMSO (AmB vehicle), or a 4:1 molar excess of AmB and EGCG was added. After blocking for 1 h at RT with 10% nonfat milk in TBS containing 0.01% Tween 20 (TBS-T), the membrane was incubated for another 1 h at RT with the polyclonal specific anti-oligomer A11 antibody (Life Technologies) or the fiber-specific monoclonal antibody OC (Millipore), diluted 1:1,000 in 3% BSA TBS-T. The membranes were then washed three times for 5 min each with TBS-T before being incubated for 1 h with anti-rabbit HRP conjugated anti-rabbit IgG (GE Healthcare) diluted 1:5,000 in 3% BSA/TBS-T at RT. After washing the membranes three times in TBS-T buffer, the blots were developed with the ECL Plus chemiluminescence kit from Amersham-Pharmacia (GE Healthcare). Prefibrillar oligomers and fibrillar species of A*β*42 were used as positive controls for A11 and OC reactivity, respectively.

In the case of full-length *Drosophila* Orb2A and Orb2B, recombinant proteins were overexpressed in *E*. *coli* BL21 (DE3) cells and purified under denaturing conditions using Ni-NTA agarose (Qiagen), according to the manufacturer’s instructions. The proteins were dialyzed against 1 M Urea, 100 mM KCl, 10 mM Na-HEPES (pH 7.6), 1 mM DTT, 0.1 mM CaCl_2_, 1 mM MgCl_2_ and 5% Glycerol containing buffer at RT. Dialysis was performed in a Slide-A-Lyzer mini Dialysis unit with a 7,000 MWCO (Pierce). For the A11 antibody (Biosource) immunoreactivity assay, 2 *μ*l of the dialysed proteins at the time indicated were spotted on Protran BA3 nitrocellulose membrane (Schleicher & Schhuell). The membranes were blocked overnight in TBS + 5% milk and then incubated with a 1:1,000 dilution of the A11 antibody for 1 h at RT. The binding of the A11 antibody was visualized with a HRP-tagged secondary antibody using Supersignal West Femto substrate (Pierce).

### Single-Cell Protein Microinjection

The COS-7 cell line was grown and maintained in supplemented DMEM with 10% (v/v) FBS (Life Technologies). The day before performing microinjections, cells were plated on a 35 mm dish at a density of 1 x 10^5^ cells per dish. Just before single-cell microinjection, Orb2A protein (2.5 *μ*M in PBS [pH 7.4]) was incubated in the presence or absence of a 4-fold molar excess (10 *μ*M) of AmB and EGCG for 100 min at 37.0°C to allow complex formation. The Orb2A:AmB:A11 ternary complex was formed by incubating the previously formed Orb2A:AmB binary complex for 3h at RT with the A11 antibody (100:1, binary complex:A11). Using a micromanipulator (Narishige), the samples were microinjected into the cytoplasm of single COS-7 cells double-blind (*n* = 100 to 200 cells per sample and repeated three times for each sample), along with fluorescein-labelled dextran (10,000 MW, Life Technologies), and fluorescence microscopy images were acquired using a CCD camera model C4742-95-12ER (Hamamatsu Photonics). Dextran, 1% DMSO, Vamp2Cyt and AmB were also microinjected as negative controls. Cell viability was monitored under an IX70 fluorescence microscope (Olympus) by counting the number of fluorescein-positive cells displaying a healthy morphology 3 h after microinjection, and this value was assigned as 100% cell viability. Over the following 3 d, the number of fluorescein-positive cells was counted every 24 h to calculate the cell survival rate. The Orb2A/ex1Htt chimeric proteins were microinjected in the same conditions. The data are represented as the mean ± SEM, using two-way ANOVA and a Bonferroni post-test, and one-way ANOVA and a Tukey post-test for statistical analysis of the time course survival curves and survival rates after 24 h, respectively. Statistical analyses were performed using GraphPad Prism 5 (GraphPad Software).

### ITC

ITC experiments to examine the interaction of full-length Orb2A with the minimal active core of the QBP1 and SCR peptides [54] were carried out in a VP-ITC microcalorimeter at 25°C. The proteins were equilibrated in PBS (pH 7.4) by gel-filtration chromatography, and the equilibration buffer was used to prepare the peptide solutions. Protein/peptide binding was tested by successive injections of the protein (10 to 25 *μ*l each) into the reaction cell loaded with peptide at a high final (peptide)/(protein) molar ratio. The apparent heat of reaction for each injection was obtained by integration of the peak area. The heat developed with the protein or peptide dilutions was determined in separate runs, loading the sample cell or the injection syringe with buffer in the conditions used for the binding experiments. The complexity of the system and the lack of precise information on the distribution of the Orb2A conformations before and after complex formation precluded the quantitative analysis of the titration curves. The protein (Orb2A, 185 *μ*M) and ligand (QBP1 and SCR, 300 *μ*M and 51 *μ*M, respectively) concentrations in the loading solutions were measured spectrophotometrically using their respective extinction coefficients.

### *Drosophila* Strains

We used the following *Drosophila* strains obtained from the Bloomington Stock Center: Elav-Gal4 (stock no. 458), Drl-Gal4 (stock no.4669), and UAS-GFP (stock no. 1522). The following transgenic lines were generated by us for the research described here: UAS-Orb2A-EGFP, UAS-Orb2B-EGFP, UAS-Orb2Δ88-EGFP, UAS-Orb2Δ162-EGFP, and UAS-Sup35NM-Orb2A-EGFP. Various genetic combinations were made by standard genetic crosses. We also used the UAS-QBP1 and UAS-SCR lines in which QPB1 and SCR were expressed at a similar level in fly heads when they were expressed under the gmr-Gal4 driver [53]. Flies expressing HttQ128 were previously described [50].

For the behavioural assays, the flies were maintained using standard fly husbandry. Briefly, flies were raised on standard cornmeal food at 25°C and 60% relative humidity on a 12 h/12 h light:dark cycle. All flies (1 d old) were collected and placed in a vial with fresh food for 24 h at 25°C prior to behavioural testing, and all transgenic lines were backcrossed at least six times with white-eyed Canton-S flies. All controls and transgenic lines carried one copy of the mini white gene known to influence fly behaviour.

### Male Courtship Suppression Assay

The male courtship conditioning assay was modified from that described previously [73]. A five-d-old male virgin was paired with a freshly mated female for three sessions of 2 h each, with a 30 min rest period in between. Memory performance was tested with a fresh-mated female 5 min and 24 h after the three sessions of spaced training. A courtship index (CI) was measured as the fraction of time the tested male spent chasing the female in a 10 min interval. The Memory Index was calculated as: $\frac{{\bar{CI}}_{Naive}-{\bar{CI}}_{Trained}}{{\bar{CI}}_{Naive}}\times 100$, where *CI*_*Naive*_ and *CI*_*Trained*_ are the mean courtship indices for independent samples of naive and trained males, respectively. GraphPad Prism version 4.0 was used for statistical analysis. We assumed statistical significance at *p < 0.05. One-way ANOVA was used for comparing memory index between each genotype.

### Heat Box Paradigm

The heat box apparatus was built in the workshop of Konrad Ochsner at the Universitiy of Wuerzburg and is a modified version of the one used by [74]. In brief, the heat chamber system consists of an array of 16 chambers (length, 29 mm; width, 4 mm; height, 2 mm) operated in parallel. Upper and counter ceilings are equipped with Peltier elements that control the temperature in the chamber. Glass side walls allow the transmission and detection of infrared light from an LED source (invisible to the flies). When a fly walks along the length of the chamber, it casts a shadow on a bar code reader (light gate array), and this signal is sent to a computer. The fly position signal from the bar code reader is sent to the computer with a frequency of 10 Hz. All experiments were conducted in complete darkness. Measurements are performed on at least three different days to avoid effects of daily variability. The experiment consists of a preference test, training and memory test. A computer controls the experiment in all three phases by continuously monitoring the time and direction of transition at the light gate. One half of the chamber is defined as the punished side and the other half as the unpunished side. These designations are switched for every experiment to avoid aftereffects of previous experiments (e.g., pheromones or stress signal). During the preference test (30 sec), the fly can explore the chamber at a constant temperature of 24°C, providing a measure of experience-independent spatial preference. In the training phase (4 min) the whole chamber is heated at 37°, whenever the fly enters the punished side and cools down to 24°C when it enters the unpunished side. In the following 3 min test phase, the temperature is kept constantly at 24°C. The performance index (PI) is calculated as the difference between the time the fly spent in the unpunished versus that in the punished side of the chamber divided by the total time. Thus, the PI can range from −1 to 1, with a PI of 0 indicating no side preference. The PI is a measure of heat avoidance and memory score during the training and memory test, respectively. To yield a measure of general activity, the sum of position changes over time is calculated. The thermosensitivity assay is recorded adjusting the temperature in the front versus back half of the chamber independently and also independent of the flies’ actions. It is stepped from 24°C on both sides to 24°C/37°C and 24°C/41°C for 1 min intervals, sequentially alternating the side with the higher temperature. The time spent on a given side is measured, and heat avoidance indices are calculated as above (PI). All animals that do not show substantial motor activity or do not experience punishment were excluded from the study.

### Additional Methods

Complete and detailed information on the cloning process for all the constructs used in this work, for the protein/polyprotein (pFS-2) sample preparation, or for protein expression and purification, can be found in [41]. A complete list of the oligonucleotides used here can be found in S2 Table.

The details of the experimental procedure and the analysis performed for the CR binding assay, far-UV CD spectroscopy, TEM imaging, 1-D ^1^H and 2-D ^1^H NOESY NMR spectroscopy, turbidimetry, and AFM-SMFS can be found elsewhere [41]. Additional information regarding FRAP and FRET can be found in [11]. The buffers, incubation times, and storage conditions, and the protein/peptide concentrations used in the aforementioned techniques, are detailed below.

#### CR binding assay

Samples were aged for 7 d at 37°C with 0.02% NaN_3_ and no stirring prior to performing the measurements. The protein samples (10 *μ*M) were dialyzed in PBS (pH 7.0), and the SCR and QBP1 peptides were used at 50 *μ*M.

#### CD spectroscopy

For far-UV CD spectra, we used a protein concentration of 2–5 *μ*M in 5 mM NaH_2_PO_4_, with 0.02% NaN_3_ (pH 6.0). The QBP1 or SCR (when used) were 5-fold molar excess relative to the proteins (final concentrations 10 to 25 *μ*M with a final concentration [v/v] of DMSO below 0.01%). After recording the first protein spectrum (time 0), the protein was incubated at 37°C without stirring, and additional spectra were collected at the times indicated.

#### Turbidimetry

Protein samples were used at 10 *μ*M in PBS + 0.02% NaN_3_ (pH 7.0) for all the kinetic experiments. The samples were incubated at 37°C without stirring, and the SCR and QBP1 peptides were used at 50 *μ*M.

#### TEM imaging

Protein samples (10 *μ*M) were dialyzed in PBS (pH 7.0) to obtain all the TEM measurements. Images were taken after 7 d of incubation at 37°C in the presence of 0.02% NaN_3_ without stirring, and the SCR and QBP1 peptides were used at 50 *μ*M.

#### NMR spectroscopy

For 1-D ^1^H and 2-D ^1^H NOESY NMR spectra recorded at 25°C, we used a protein concentration of 150–300 *μ*M in NaH_2_PO_4_ (pH 6.6–6.9) with 10% D_2_O and 50 *μ*M DSS in some duplicate samples as the internal chemical shift reference.

#### AFM-SMFS

Experiments were performed using 2–3 *μ*M polyproteins in 10 mM Tris-HCl (pH 7.5) at RT with proteins incubated at 4°C between sessions and using NTA-Ni^2+^ functionalized coverslips. For experiments in the presence of the QBP1 and SCR peptides, we used 20 *μ*M of the peptide dissolved in DMSO and the samples were incubated overnight with the peptide at 4°C before taking measurements.

## Supporting Information

S1 Data Raw data from the plots shown in figures. (XLSX)

S1 Fig ThT analysis and the oligomeric states of Orb2. **(A)** Nine purified recombinant proteins were used as controls to assess the specificity of the ThT binding assay under our experimental conditions. The A*β*42 peptide was used as a positive control in the assay of ThT fluorescence emission. **(B)** Chemicals known to dissociate or prevent amyloid-like aggregates inhibit Orb2A-ThT reactivity. Recombinant Orb2A was purified under denaturing conditions, and it was then allowed to refold in the presence of EGCG, rottlerin, or chloroquinone. EGCG and rottlerin very effectively blocked Orb2A amyloid formation. Surprisingly, chloroquinone, which is effective against mammalian PrP amyloid had almost no effect on Orb2A oligomerization. **(C)** Orb2A forms amyloids more efficiently than the canonical prion-like protein Sup35NM or the Alzheimer’s A*β*42 peptide. **(D)** The Orb2A protein´s ability to adopt the prion-like state correlates with monomeric and SDS-Urea resistant oligomeric protein states. Orb2 colonies were grown to mid log phase, and the supernatant (S) and pellet (P) fractions were analyzed in 1.5% SDS-agarose gels following 12,000 × g centrifugation. Consistent with its prion-like behaviour (see Fig 1H), the proportion of Orb2A in the pellet fraction was higher, and it formed more SDS-resistant oligomers than Orb2B. The underlying data for panels in this figure can be found in S1 Data.
(TIF)

S2 Fig Insertion of the TEV target sequence does not alter the physical properties of Orb2. **(A)** Orb2A with TEV protease sites inserted at the indicated positions and wild type Orb2A form similar puncta in adult ring neurons. Scale bar: 25 *μ*m. **(B)** Similar distribution of Orb2A (top) and Orb2B (bottom), with or without the TEV protease recognition sequence, in the supernatant (top) and pellet (bottom) fractions. **(C)** Insertion of the TEV protease recognition sequence does not alter the RNA binding activity of Orb2A. Orb2A wild type and Orb2A carrying the TEV protease recognition sequence bind similarly to Oskar mRNA. * indicates a cross reacting protein. **(D)** The TEV recognition sequence located outside the PLD (Orb2A216TEV) can be cleaved by the TEV protease, but it is resistant to the protease when inserted within the PLD (Orb2A88TEV). S2 cells were transfected with Orb2A or Orb2B constructs carrying the 3xFLAG-tagged TEV protease under a metallothionein promoter. The expression of the TEV protease was induced by adding 0.7 mM CuSO_4_, and the cells were analyzed 12 h after induction. The punctuate appearance of Orb2AEGFP does not depend on protease expression when the TEV site was within the PLD (Orb2A88TEV), both in the presence or absence of the TEV protease. However, when the TEV lies outside the PLD (Orb2A216TEV), the C-terminus of Orb2A carrying the EGFP tag is cleaved in the presence of TEV protease, and the punctate appearance of Orb2A is no longer evident. Interestingly, upon cleavage of the 216 N-terminal residues of Orb2A and 370 residues in Orb2B, the EGFP-tagged C-terminus moves into the nucleus. Scale bar: 5 *μ*m.
(TIF)

S3 Fig The PLD can adopt distinct protease-accessible states in its monomeric form. The studies of adult brain lysates suggested that the monomeric Orb2 proteins may also have distinct protease accessibilities. To test this, S2 cell extracts were first clarified by centrifugation at 10,000 × g to remove the large aggregates. The supernatant was centrifuged at 174,000 × g to obtain the supernatant and pellet fractions, and each fraction was treated with the TEV protease in a high salt solution. The N- and C-terminal fragments obtained by TEV protease cleavage are indicated. The TEV target sequence located inside the PLD of the Orb2A monomer in the pellet fraction (Orb2A88TEV or Orb2A216TEV) is inaccessible, whereas it is partly accessible in the monomer form that was found in the supernatant (Sup). When the TEV is located outside the Orb2A PLD, it is equally accessible in the supernatant and the pellet fraction (Orb2A216TEV). The N-terminal fragment of Orb2A (red) derived from the pellet fraction is not recognized by Ab273 (middle panel) but it is recognized by Ab2233, whereas both antibodies recognize the same fragment that partitions to the supernatant. When the TEV site is in the Orb2B PLD (Orb2B242TEV), it was partially accessible to the protease, while in Orb2B from the supernatant and pellet fractions it was fully accessible when the TEV site lies outside the PLD (Orb2B370TEV). Unlike Orb2A, in Orb2B the N-terminal fragment derived from both the pellet and supernatant fractions is recognized by Ab273 and Ab2233.
(TIF)

S4 Fig The effect of the F5Y mutation on amyloid-like oligomer formation by Orb2A. **(A)** The aggregation of the Orb2AF5Y PLD mutant was drastically reduced. **(B)** The far-UV CD spectra of freshly dissolved Orb2A PLD and F5Y variant are very similar. Upon aging, the loss of signal due to protein precipitation is much greater for wild type Orb2A PLD than the F5Y variant. **(C)** The F5Y mutation reduces amyloid formation, as assessed by measuring the concentration of bound CR dye. The data represent the mean ± SEM: ***p < 0.001 (One-way ANOVA and Tukey post-test). **(D)** A representative TEM micrograph shows that the presence of oligomers and amyloid fibers was drastically reduced by the F5Y mutation. Scale bar: 1 *μ*m. The underlying data for panels in this figure can be found in S1 Data.
(TIF)

S5 Fig Orb2 PLD does not alter the fold of the carrier module upon insertion for SMFS analysis. **(A)** Orb2A sequence was computationally evaluated by Rossetta and PONDR-FIT algorithms, showing specific residues (blue & numbered for Rossetta analysis of Orb2A PLD) predicted to form amyloid fibers and regions predicted to be ordered/disordered (red, PONDR-FIT), respectively. **(B)** Schematic representation of the I27-Orb2A PLD carrier-guest construction prepared by VMD 1.8.6 [75], using atomic coordinates for titin I27 (grey, PDB code 1TIT) and off-template modelling for Orb2A PLD (dark yellow). **(C)** The far-UV CD spectra of I27-Orb2A PLD, I27-MCS and the Orb2A PLD. The close similarity of the I27-Orb2A spectrum to the sum of I27-MCS and Orb2A PLD spectra, weighted to take into account their different number of residues, strongly suggests that the Orb2A PLD behaves like an isolated domain and remains disordered when grafted into the I27 carrier. **(D)** The Orb2A PLD ^1^H NMR spectrum (red) has resonances with chemical shift values typical of a random coil. Compared to the spectrum of I27 that carries a small loop resulting from the translation of the multicloning site (I27-MCS, black), in the I27-Orb2A PLD spectrum (blue) the signals of native I27 are retained but somewhat broadened due to the slower tumbling, indicating that I27 remains folded while Orb2A PLD is still disordered. **(E)** Left panel: 2-D ^1^H NOESY spectrum of I27 (black) showing the downfield region. Some resonances are labelled that are representative of folded I27, such as Ala 82 and Leu 84 in the "mechanical clamp," or Trp 34 and Ile 23 in the hydrophobic core. Right panel: view of the 2-D ^1^H NOESY spectra of I27 alone (black) and hosting Orb2A PLD (blue) to show the correlations resulting from contact between aromatic and aliphatic hydrogen nuclei in the hydrophobic core. The observation of these peaks in the 1D ^1^H spectra of I27 hosting Orb2A PLD (see S5D Fig), and their close similarity to the published chemical shift values [76], indicates that I27 maintains its structure while hosting Orb2A PLD. The underlying data for panels in this figure can be found in S1 Data.
(TIF)

S6 Fig Orb2A PLD preserves the amyloidogenic properties in the fusion protein. **(A)** Aggregation of both isolated and carrier-fused Orb2A PLD over the incubation period monitored by turbidimetry at 405 nm. **(B)** Concentration (*μ*M) of CR bound to the amyloid structures formed by both isolated and carrier-fused Orb2A PLD samples. The data are represented as the mean ± SEM: ***p < 0.001 (One-way ANOVA and Tukey post-test). **(C)** A representative electron micrograph of aged I27-Orb2A PLD shows oligomers (asterisks) and amyloid fibers (arrows) resembling those of the full-length Orb2A (Fig 1G) or Orb2A PLD (Fig 4C). Scale bar: 1 *μ*m. The underlying data for panels in this figure can be found in S1 Data.
(TIF)

S7 Fig Nanomechanics of Orb2A PLD obtained with the “protection strategy.” **(A)** Schematic cartoon representation of the pFS-2+Orb2A PLD construction prepared with MODELLER and displayed by VMD 1.8.6 [75]. The polyprotein encoded by the pFS-2 vector also contains a random coil region (a fragment of titin N2B, in grey) that acts as a spacer to avoid the noisy proximal region of the force-extension recordings, a major problem in AFM-SMFS [77]. The structural model was generated by aligning the ends of multiple ubiquitin structures (in black, PDB code 1UBQ), and the off-template generated structures for N2B (grey) and Orb2A PLD (dark yellow). **(B)** Representative force-extension recordings of pFS-2+Orb2A PLD using the “protection strategy” based on the pFS-2 vector [77]. This approach revealed different conformations adopted by the Orb2A PLD, from the NM (in yellow, left column) or M (in red, right column) conformations, showing different *F* and Δ*L*_*c*_ values, and suggesting rich conformational polymorphism of this PLD at the monomer level. The elasticity of the stretched proteins was analyzed using the WLC model of polymer elasticity [78]. The carrier module (in grey) must unfold completely (“a” is Δ*L*_*c*_ for it) before the force can access the guest Orb2A PLD: “b” and “c” represent the Δ*L*_*c*_ for NM and M regions of Orb2A PLD, respectively; and b + c corresponds to the complete length of the stretched Orb2A PLD. The complete extension of Orb2A PLD is 64.8 nm (162 residues x 0.4 nm/residue), and that of I27 is 29.5 nm.
(TIF)

S8 Fig Additional AFM-SMFS analysis of Orb2A PLD and Orb2AF5Y PLD. **(A)** The average number of force peaks (structures) per molecule is shown, where 0 represents the NM conformations. The M conformers usually show more than one force peak per Orb2A PLD molecule, suggesting the formation of several mechanical barriers at different positions of the molecule. **(B)** Scatter plots show that, in the M conformers, there is no clustering between *F* and Δ*L*_*c*_, which suggests that no preferred structures are formed. **(C)** The recording (top) represents an example of an incomplete or “putative” M event, in which we assume that the polyprotein attachments (either from the tip or the substrate) were lost before the molecule was fully stretched. Therefore, it is reasonable to assume that its *F* value would be higher than the detachment force (indicated by the arrow). The spectrum shows the force peaks due to the unfolding of the I27 carrier and a region of the Orb2A PLD that is shorter than the expected full-length protein (61 nm versus 94.3 nm from I27-Orb2A PLD complete unfolding). *F* detachment versus # of putative events analysis (bottom) suggests that the population of M conformers actually observed in our experiments is an underestimation. The presence of putative data (i.e., incomplete force-extension runs) strongly indicates that the true population of M conformers is probably more abundant than that observed. Putative data were not included in our sample size (n). **(D)** The average number of force peaks (structures) per molecule is decreased in the F5Y variant. **(E)** No evidence for the preferential formation of specific structures was obtained by SMFS of pFS-2+Orb2AF5Y PLD. **(F)** Putative incomplete recordings are fewer and of lower mechanical stability in the presence of the F5Y mutation suggesting the formation of fewer, weaker M conformers. The underlying data for panels in this figure can be found in S1 Data.
(TIF)

S9 Fig Additional results from the protein microinjection assay. **(A)** Negative controls do not cause cell death after microinjection. **(B)** Survival of COS-7 cells microinjected with several samples after 24 h (***p < 0.001, one-way ANOVA and Tukey post-test). The underlying data for panels in this figure can be found in S1 Data.
(TIF)

S10 Fig Domain swapping of Orb2 with an expanded exon 1 of Huntingtin produces a toxic phenotype. **(A)** Pictograms showing the domain organization of Orb2A, ex1HttQ72, and the chimeric proteins. **(B)** Structural features during the assembly of expanded exon 1 of Huntingtin (ex1HttQ72) and the chimeric constructions (ex1Htt-Orb2APLD-ex1Htt and ex1HttQ72-Orb2A C-terminal) probed with the conformational antibody A11 showed the formation of more stable species reactive to A11 for ex1HttQ72 and ex1HttQ72-Orb2A C-terminal. **(C)** Survival curves of COS-7 cells microinjected with the chimeric proteins (their A11-reactivity at microinjection time is indicated in an inset). Data are represented as the mean ± SEM: ***p < 0.001 (blue asterisks, ex1Htt-Orb2APLD-ex1Htt versus ex1HttQ72 and ex1HttQ72-Orb2A C-terminal; Two-way ANOVA and Bonferroni post-test). The number of single-cells microinjected per sample was *n* = 100–200. **(D)** Fluorescence micrographs of COS-7 cells microinjected with ex1HttQ72, ex1Htt-Orb2APLD-ex1Htt, and ex1HttQ72-Orb2A C-terminal at 0 and 24 h after microinjection. Microinjection of ex1HttQ72 and ex1HttQ72-Orb2A C-terminal resulted in a marked drop in the number of live cells at 24 h compared to those at 0 h, while ex1Htt-Orb2APLD-ex1Htt did not exhibit cytotoxicity. Scale bars: 100 *μ*m. The underlying data for panels in this figure can be found in S1 Data.
(TIF)

S11 Fig Additional results from Orb2-HttQ128 hetero-oligomers. **(A)** HttQ128 forms larger fluorescence puncta that specifically recruit Orb2A to hetero-aggregates, whereas synaptic proteins like synaptotagmin or GRASP-65 are not recruited **(B)**. **(C)** Replacing the Orb2A PLD with Sup35NM results in the formation of HttQ-Sup35NMOrb2AC hybrid oligomers. Scale bar: 20 *μ*m.
(TIF)

S12 Fig Additional AFM-SMFS analysis of Orb2A PLD in the presence of QBP1. **(A)** The QBP1 peptide reduces the number of force peaks per molecule, suggesting that it decreases the propensity of Orb2A PLD to adopt *β*-structures (numbers in the inset are the average number of force peaks per Orb2A molecule). **(B)** Incomplete recordings of putative M events decrease in the presence of QBP1 inhibitor peptide, which suggests the formation of fewer, and weaker, M conformers. **(C)** No preferential structures were detected when stretching pFS-2+Orb2A PLD in the presence of QBP1 (left panel) or the SCR (right panel). The underlying data for panels in this figure can be found in S1 Data.
(TIF)

S13 Fig Expression of QBP1 interferes with long-term male courtship suppression. CI of male flies that were exposed to an unreceptive female was compared to that of naïve, untrained male flies. Pan-neuronal expression of QBP1 using the Elav-Gal4 driver interferes with long-term but not short-term memory, while the SCR control peptide has no effect (see Fig 7). The genetic controls for each group were tested simultaneously, and the number of flies used in each group is indicated. The underlying data for panels in this figure can be found in S1 Data.
(TIF)

S14 Fig Transient expression of QBP1 does not interfere with long-term memory. The QBP1 peptide was expressed in the adult nervous system 1 d prior to the behavioral training using the RU486-inducible GeneSwitch-Gal4 system. The 3–4 d old flies were fed with 1 mM RU486 for 24 h to induce the expression of QBP1. Following drug feeding the flies were trained in the male courtship suppression paradigm and memory was measured at 24 h. Both control (-RU486) and experimental groups showed significant courtship suppression at 24 h, suggesting that long-term memory was not affected by the transient expression of QBP1. The number of flies tested in various groups are indicated. *p < 0.05 (unpaired two-tailed Student’s *t* test). The underlying data for panels in this figure can be found in S1 Data.
(TIF)

S15 Fig QBP1 expression does not interfere with short-term avoidance memory. **Top**. Schematic of the single training protocol in the heat box operant conditioning paradigm. In this operant conditioning paradigm, individual flies were conditioned to avoid one side of a uniform chamber [74]. Each time the fly enters the predetermined “punish” side of the chamber, the temperature of the entire chamber is heated to 37°C and the temperature is brought back to 24°C when the fly moves to the other “unpunished” side. Following training, the flies learn to avoid the punish side even in the absence of punishment. Memory is measured as the duration of the preferred response for unpunished side. Flies are either trained in a single trial protocol or multiple trial protocol where the single training protocol is repeated successively the indicted times. **Bottom**. The avoidance index after each training session. With repeated training, the avoidance index increases, and after six training sessions the performance plateaus. There was no significant difference in the performance between wild type flies and flies expressing QBP1 chronically in the nervous system. The underlying data for panels in this figure can be found in S1 Data.
(TIF)

S1 Table Summary of the AFM-SMFS analysis. NM, Nonmechanostable. M, mechanostable. SEM was calculated assuming the M and NM as states from a Bernoulli variable. The values are only shown in the NM column since they are the same as for the M states. Putative data were not included in our sample size (n).
(TIF)

S2 Table Summary of the oligonucleotides used. The restriction sites introduced by PCR into the amplified sequences are in italics. The extra sequences added to each restriction site were recommended by New England Biolabs to enhance the digestion efficiency of linear DNA sequences. The vectors and strategy used are the same as those described previously [41].
(TIF)

## Abbreviations

AFM-SMFS: atomic force microscopy-based single molecule force spectroscopy
AmB: amphotericin B
ApCPEB: *Aplysia* CPEB
CD: Circular Dichroism
CFP: cyan fluorescent protein
CI: courtship index
CPEB: cytoplasmic polyadenylation element binding protein
CR: Congo red
DMSO: dimethylsulfoxide
DSS: sodium 4,4-dimethyl-4-silapentane-1-sulfonate
EFF-1: epithelial fusion failure 1
EGCG: epigallocatechin gallate
EGFP: enhanced green fluorescent protein
FRAP: fluorescence recovery after photobleaching
FRET: fluorescence resonance energy transfer
FTIR: Fourier transform infrared
HttQ128: Huntingtin protein-containing expanded polyQ repeats
ITC: isothermal titration calorimetry
M: mechanically resistant
NM: nonmechanically resistant
NMR: nuclear magnetic resonance
NOESY: nuclear Overhauser effect spectroscopy
PI: performance index
PLD: prion-like domain
QBP1: polyglutamine-binding peptide 1
Q/N: glutamine, asparagine
SDS: sodium dodecyl sulphate
SEM: standard error of the mean
TBS: Tris-buffered saline
TEM: transmission electron microscopy
TEV: tobacco etch virus
ThT: thioflavin T
WLC: worm-like chain
YPD: yeast extract peptone dextrose
