# Supplementary figures and images for: Molecular Basis of Orb2 Amyloidogenesis and Blockade of Memory Consolidation
Source: PLoS Biol. 2016 Jan 26;14(1):e1002361. doi: 10.1371/journal.pbio.1002361 (PMC4727891; doi:10.1371/journal.pbio.1002361)

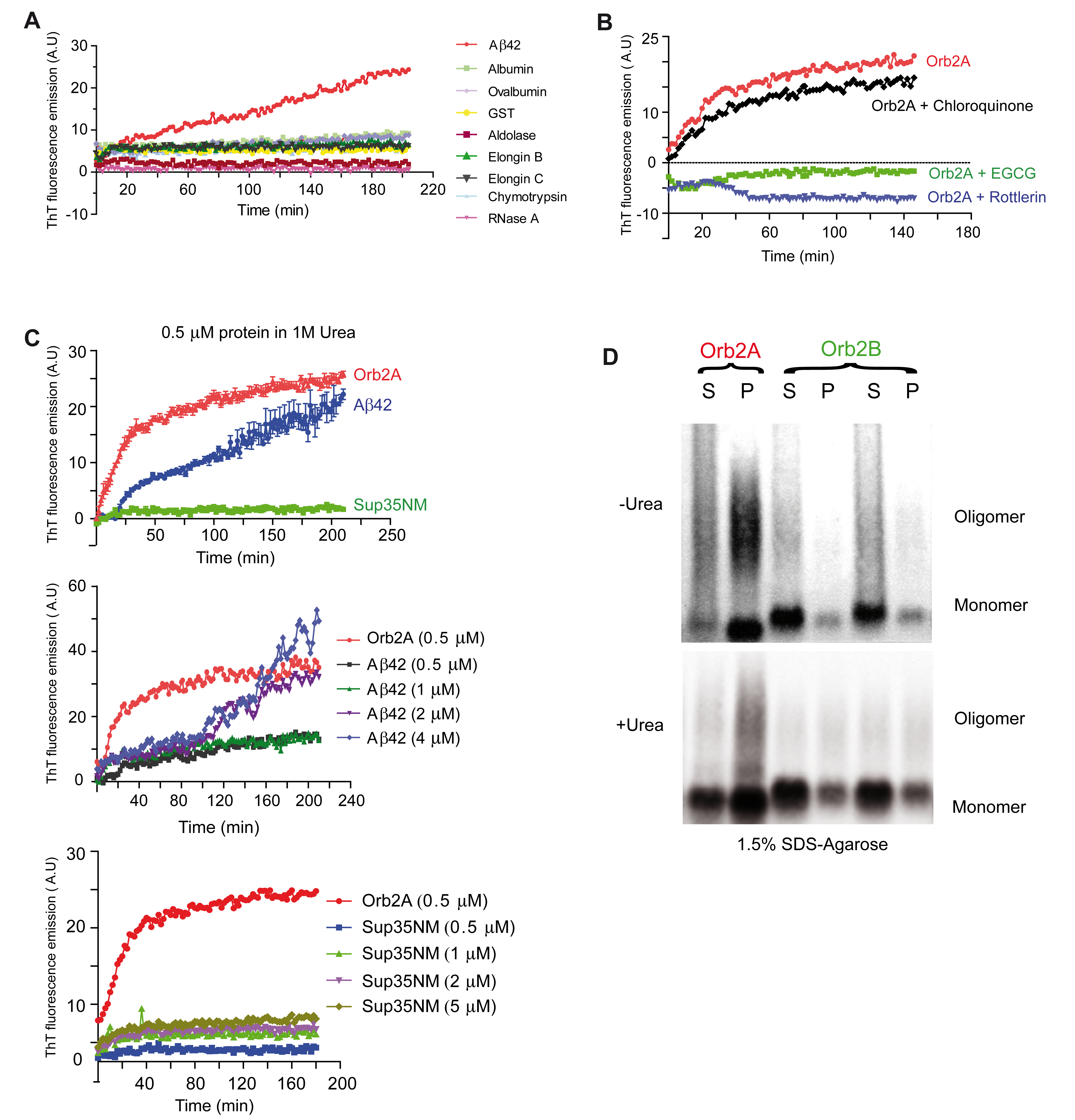

Supplement: S1 Fig — (A) Nine purified recombinant proteins were used as controls to assess the specificity of the ThT binding assay under our experimental conditions. The Aβ42 peptide was used as a positive control in the assay of ThT fluorescence emission. (B) Chemicals known to dissociate or prevent amyloid-like aggregates inhibit Orb2A-ThT reactivity. Recombinant Orb2A was purified under denaturing conditions, and it was then allowed to refold in the presence of EGCG, rottlerin, or chloroquinone. EGCG and rottlerin very effectively blocked Orb2A amyloid formation. Surprisingly, chloroquinone, which is effective against mammalian PrP amyloid had almost no effect on Orb2A oligomerization. (C) Orb2A forms amyloids more efficiently than the canonical prion-like protein Sup35NM or the Alzheimer’s Aβ42 peptide. (D) The Orb2A protein´s ability to adopt the prion-like state correlates with monomeric and SDS-Urea resistant oligomeric protein states. Orb2 colonies were grown to mid log phase, and the supernatant (S) and pellet (P) fractions were analyzed in 1.5% SDS-agarose gels following 12,000 × g centrifugation. Consistent with its prion-like behaviour (see Fig 1H), the proportion of Orb2A in the pellet fraction was higher, and it formed more SDS-resistant oligomers than Orb2B. The underlying data for panels in this figure can be found in S1 Data. (TIF) [file pbio.1002361.s002.tif]

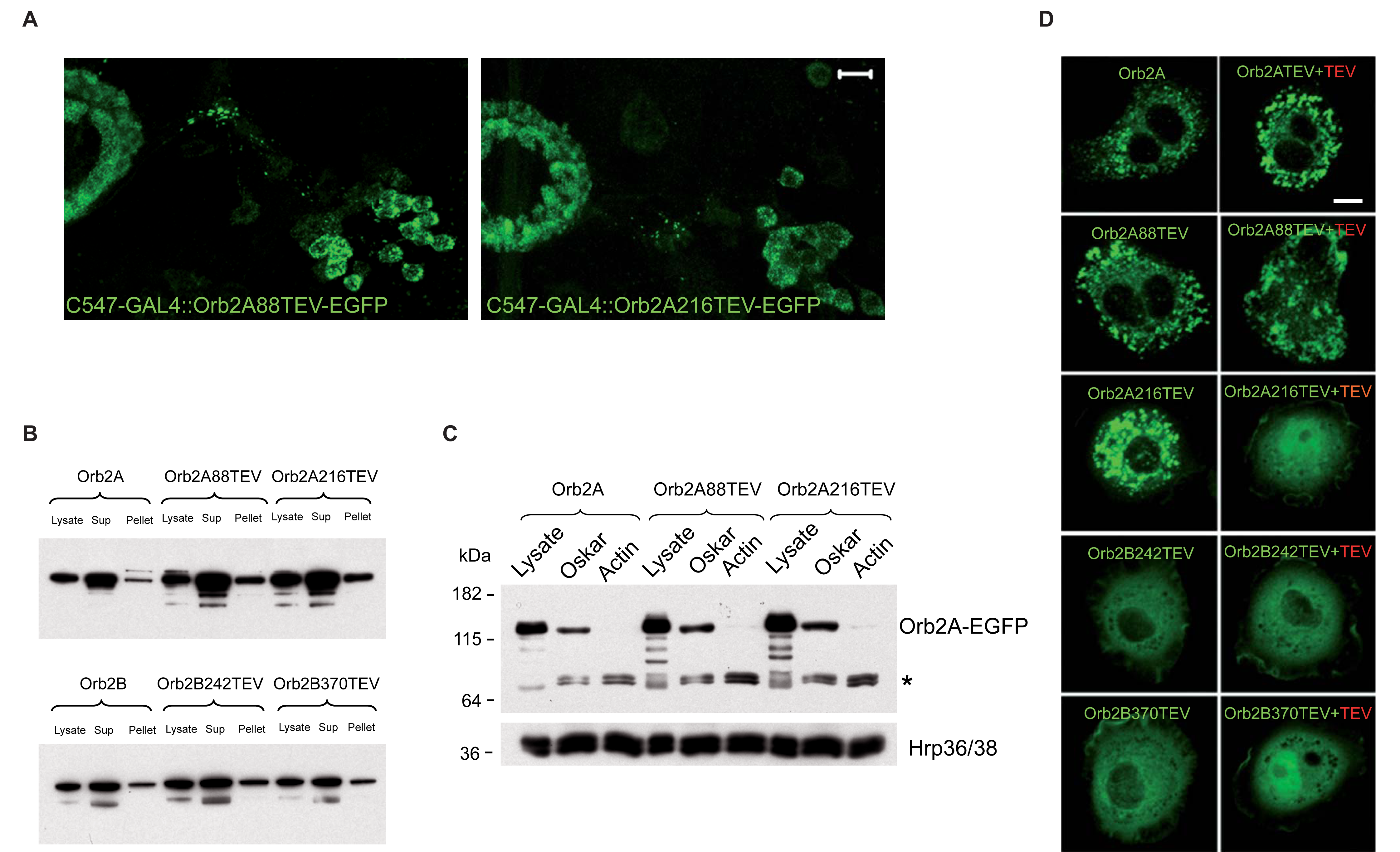

Supplement: S2 Fig — (A) Orb2A with TEV protease sites inserted at the indicated positions and wild type Orb2A form similar puncta in adult ring neurons. Scale bar: 25 μm. (B) Similar distribution of Orb2A (top) and Orb2B (bottom), with or without the TEV protease recognition sequence, in the supernatant (top) and pellet (bottom) fractions. (C) Insertion of the TEV protease recognition sequence does not alter the RNA binding activity of Orb2A. Orb2A wild type and Orb2A carrying the TEV protease recognition sequence bind similarly to Oskar mRNA. * indicates a cross reacting protein. (D) The TEV recognition sequence located outside the PLD (Orb2A216TEV) can be cleaved by the TEV protease, but it is resistant to the protease when inserted within the PLD (Orb2A88TEV). S2 cells were transfected with Orb2A or Orb2B constructs carrying the 3xFLAG-tagged TEV protease under a metallothionein promoter. The expression of the TEV protease was induced by adding 0.7 mM CuSO4, and the cells were analyzed 12 h after induction. The punctuate appearance of Orb2AEGFP does not depend on protease expression when the TEV site was within the PLD (Orb2A88TEV), both in the presence or absence of the TEV protease. However, when the TEV lies outside the PLD (Orb2A216TEV), the C-terminus of Orb2A carrying the EGFP tag is cleaved in the presence of TEV protease, and the punctate appearance of Orb2A is no longer evident. Interestingly, upon cleavage of the 216 N-terminal residues of Orb2A and 370 residues in Orb2B, the EGFP-tagged C-terminus moves into the nucleus. Scale bar: 5 μm. (TIF) [file pbio.1002361.s003.tif]

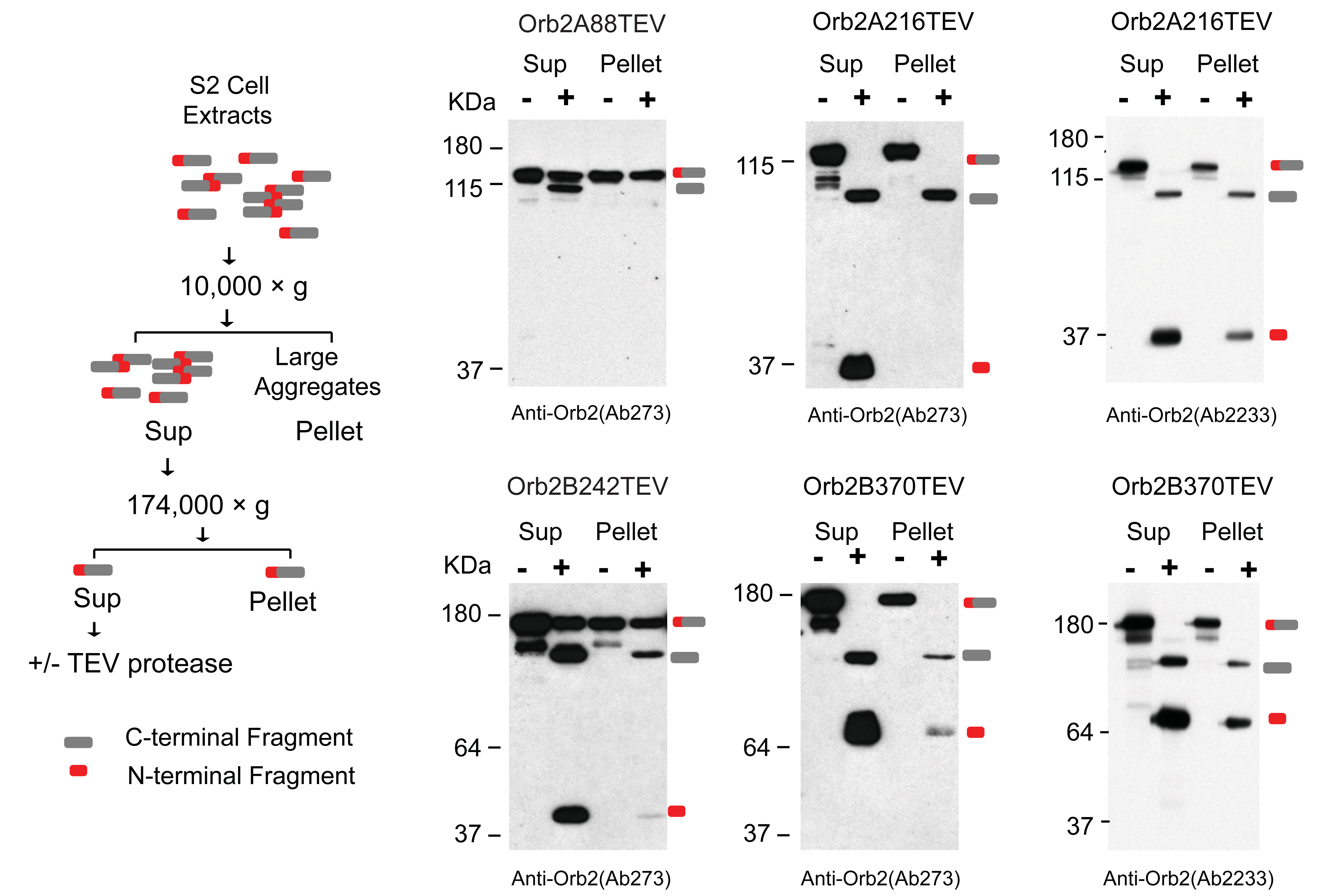

Supplement: S3 Fig — The studies of adult brain lysates suggested that the monomeric Orb2 proteins may also have distinct protease accessibilities. To test this, S2 cell extracts were first clarified by centrifugation at 10,000 × g to remove the large aggregates. The supernatant was centrifuged at 174,000 × g to obtain the supernatant and pellet fractions, and each fraction was treated with the TEV protease in a high salt solution. The N- and C-terminal fragments obtained by TEV protease cleavage are indicated. The TEV target sequence located inside the PLD of the Orb2A monomer in the pellet fraction (Orb2A88TEV or Orb2A216TEV) is inaccessible, whereas it is partly accessible in the monomer form that was found in the supernatant (Sup). When the TEV is located outside the Orb2A PLD, it is equally accessible in the supernatant and the pellet fraction (Orb2A216TEV). The N-terminal fragment of Orb2A (red) derived from the pellet fraction is not recognized by Ab273 (middle panel) but it is recognized by Ab2233, whereas both antibodies recognize the same fragment that partitions to the supernatant. When the TEV site is in the Orb2B PLD (Orb2B242TEV), it was partially accessible to the protease, while in Orb2B from the supernatant and pellet fractions it was fully accessible when the TEV site lies outside the PLD (Orb2B370TEV). Unlike Orb2A, in Orb2B the N-terminal fragment derived from both the pellet and supernatant fractions is recognized by Ab273 and Ab2233. (TIF) [file pbio.1002361.s004.tif]

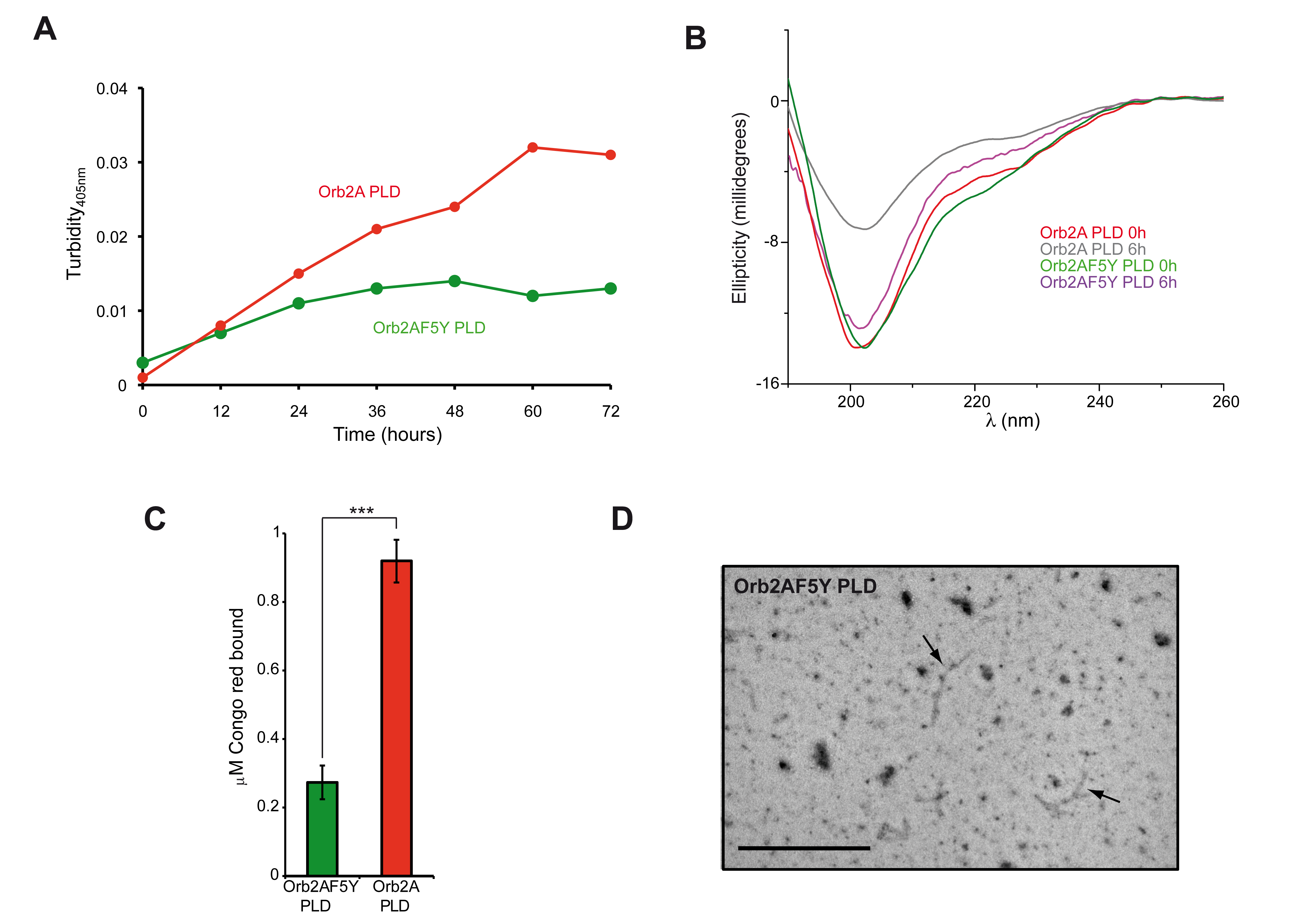

Supplement: S4 Fig — (A) The aggregation of the Orb2AF5Y PLD mutant was drastically reduced. (B) The far-UV CD spectra of freshly dissolved Orb2A PLD and F5Y variant are very similar. Upon aging, the loss of signal due to protein precipitation is much greater for wild type Orb2A PLD than the F5Y variant. (C) The F5Y mutation reduces amyloid formation, as assessed by measuring the concentration of bound CR dye. The data represent the mean ± SEM: ***p < 0.001 (One-way ANOVA and Tukey post-test). (D) A representative TEM micrograph shows that the presence of oligomers and amyloid fibers was drastically reduced by the F5Y mutation. Scale bar: 1 μm. The underlying data for panels in this figure can be found in S1 Data. (TIF) [file pbio.1002361.s005.tif]

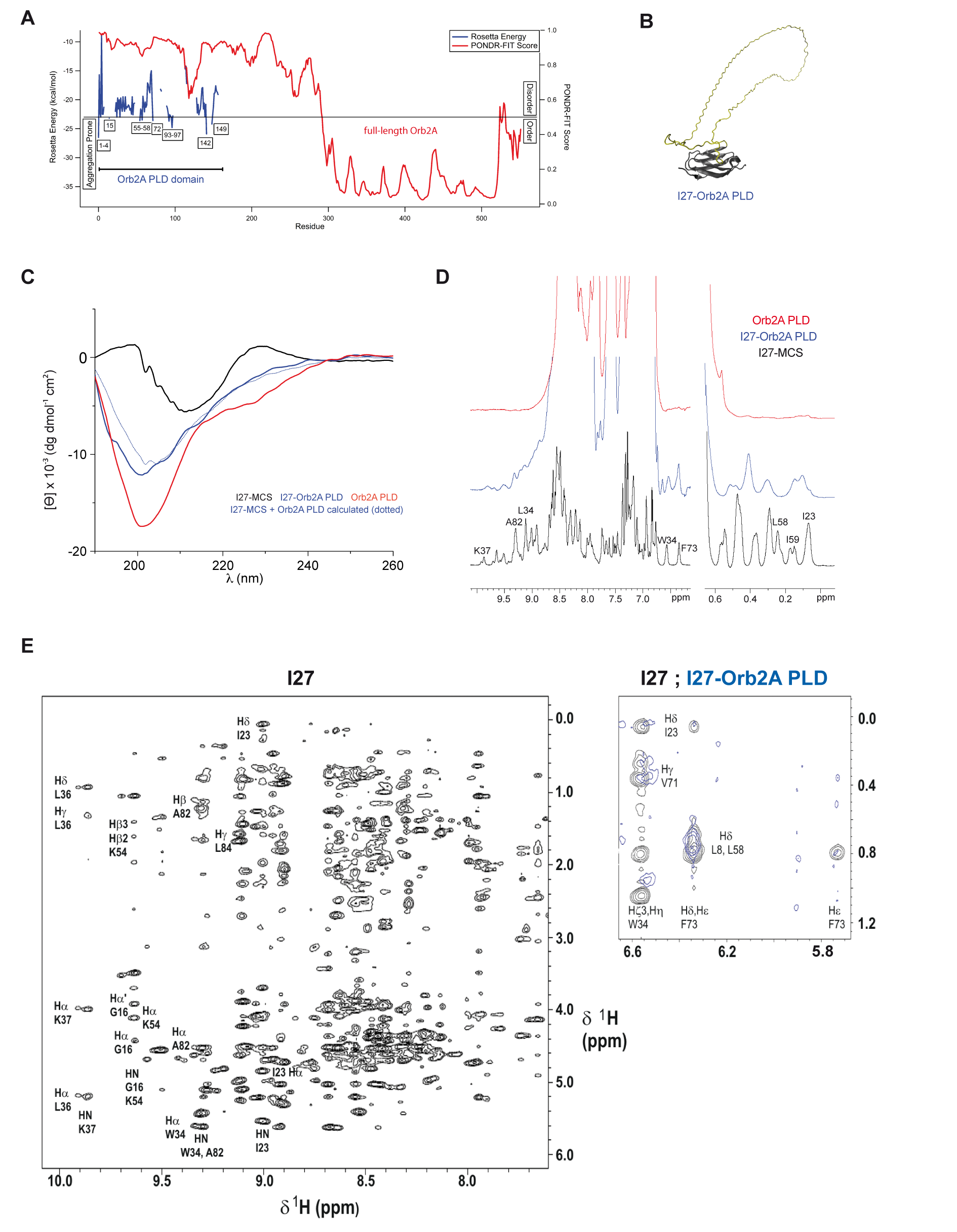

Supplement: S5 Fig — (A) Orb2A sequence was computationally evaluated by Rossetta and PONDR-FIT algorithms, showing specific residues (blue & numbered for Rossetta analysis of Orb2A PLD) predicted to form amyloid fibers and regions predicted to be ordered/disordered (red, PONDR-FIT), respectively. (B) Schematic representation of the I27-Orb2A PLD carrier-guest construction prepared by VMD 1.8.6 [75], using atomic coordinates for titin I27 (grey, PDB code 1TIT) and off-template modelling for Orb2A PLD (dark yellow). (C) The far-UV CD spectra of I27-Orb2A PLD, I27-MCS and the Orb2A PLD. The close similarity of the I27-Orb2A spectrum to the sum of I27-MCS and Orb2A PLD spectra, weighted to take into account their different number of residues, strongly suggests that the Orb2A PLD behaves like an isolated domain and remains disordered when grafted into the I27 carrier. (D) The Orb2A PLD 1H NMR spectrum (red) has resonances with chemical shift values typical of a random coil. Compared to the spectrum of I27 that carries a small loop resulting from the translation of the multicloning site (I27-MCS, black), in the I27-Orb2A PLD spectrum (blue) the signals of native I27 are retained but somewhat broadened due to the slower tumbling, indicating that I27 remains folded while Orb2A PLD is still disordered. (E) Left panel: 2-D 1H NOESY spectrum of I27 (black) showing the downfield region. Some resonances are labelled that are representative of folded I27, such as Ala 82 and Leu 84 in the "mechanical clamp," or Trp 34 and Ile 23 in the hydrophobic core. Right panel: view of the 2-D 1H NOESY spectra of I27 alone (black) and hosting Orb2A PLD (blue) to show the correlations resulting from contact between aromatic and aliphatic hydrogen nuclei in the hydrophobic core. The observation of these peaks in the 1D 1H spectra of I27 hosting Orb2A PLD (see S5D Fig), and their close similarity to the published chemical shift values [76], indicates that I27 maintains its structure while hosting Orb2A PLD. The und [file pbio.1002361.s006.tif]

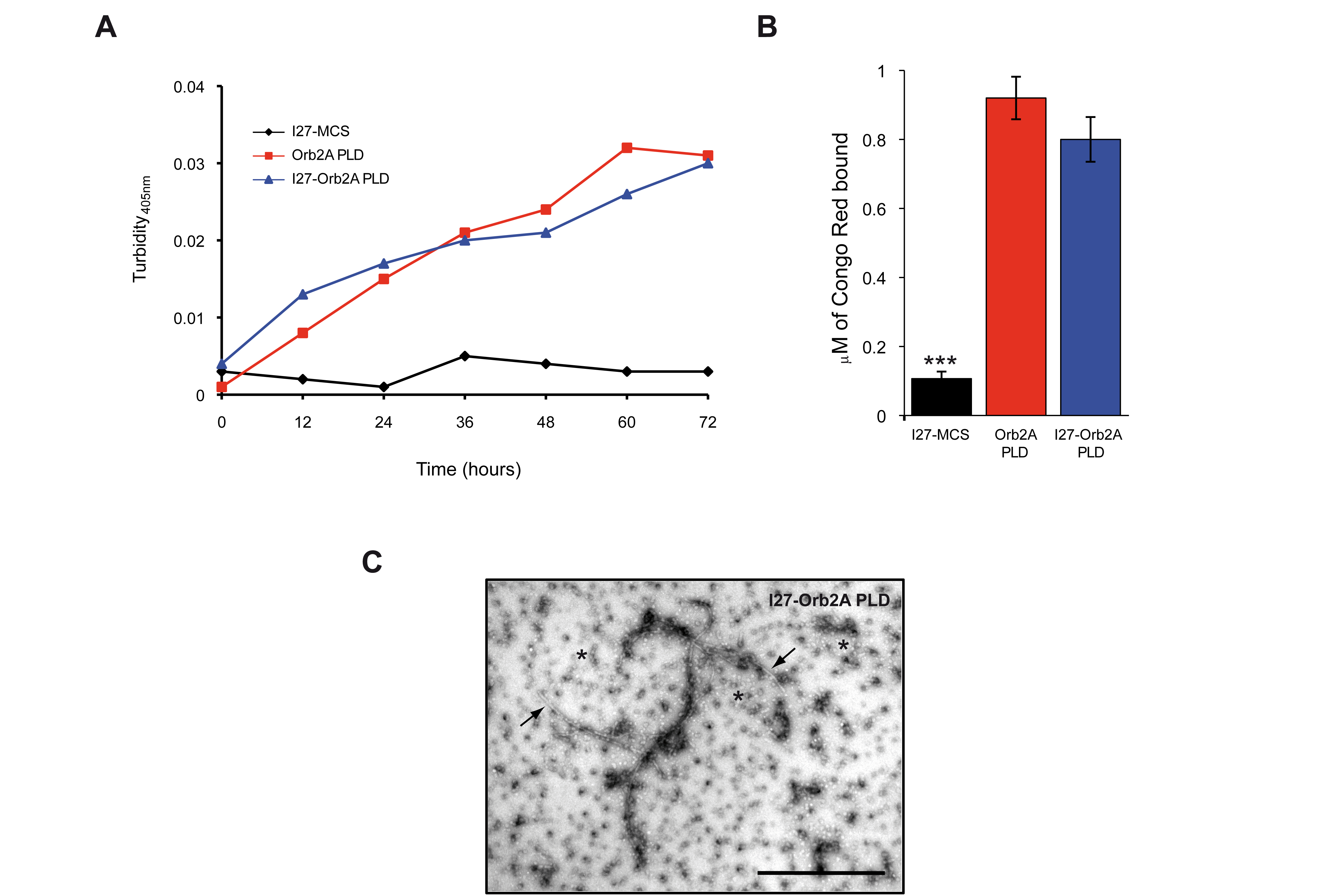

Supplement: S6 Fig — (A) Aggregation of both isolated and carrier-fused Orb2A PLD over the incubation period monitored by turbidimetry at 405 nm. (B) Concentration (μM) of CR bound to the amyloid structures formed by both isolated and carrier-fused Orb2A PLD samples. The data are represented as the mean ± SEM: ***p < 0.001 (One-way ANOVA and Tukey post-test). (C) A representative electron micrograph of aged I27-Orb2A PLD shows oligomers (asterisks) and amyloid fibers (arrows) resembling those of the full-length Orb2A (Fig 1G) or Orb2A PLD (Fig 4C). Scale bar: 1 μm. The underlying data for panels in this figure can be found in S1 Data. (TIF) [file pbio.1002361.s007.tif]

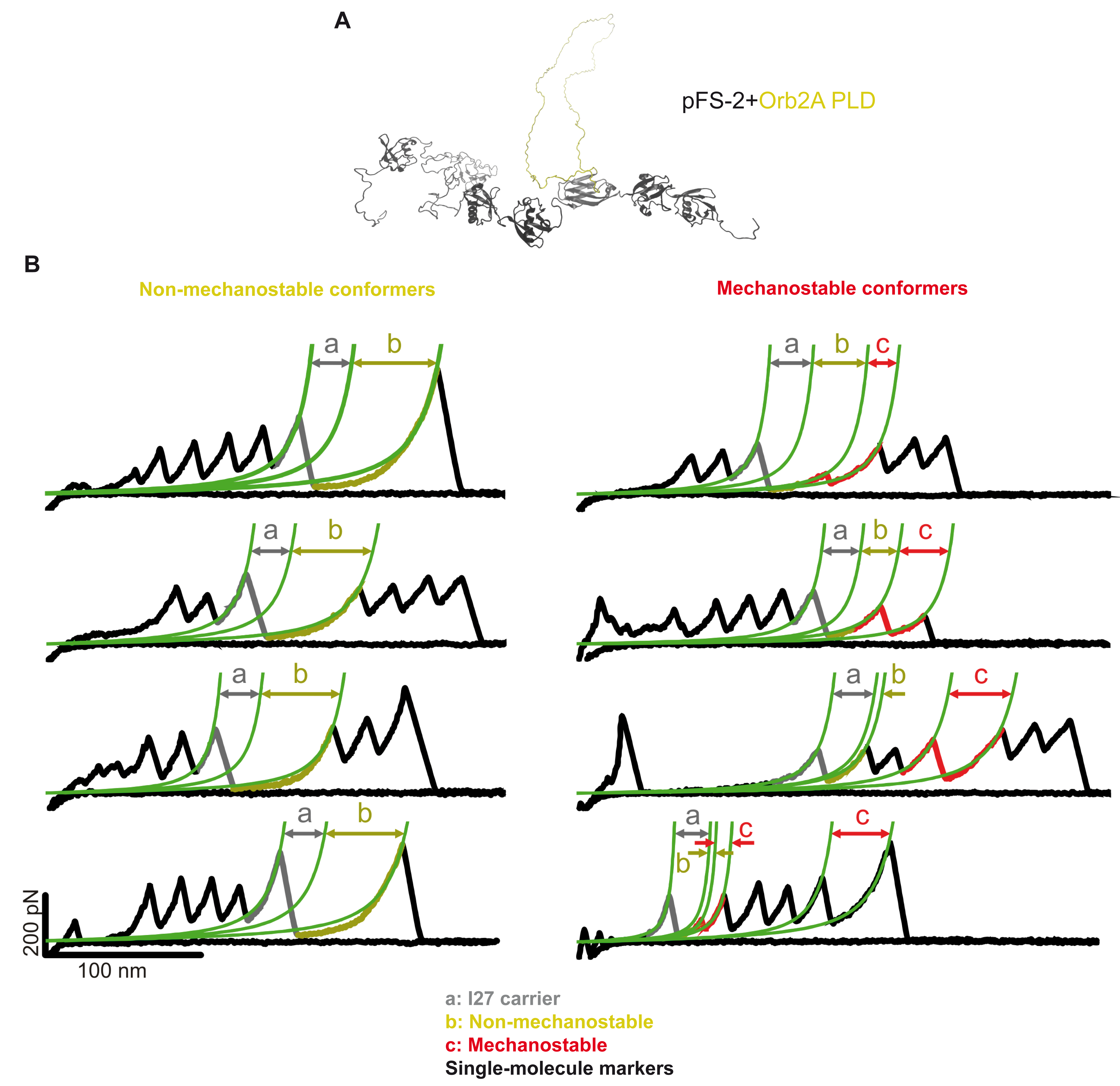

Supplement: S7 Fig — (A) Schematic cartoon representation of the pFS-2+Orb2A PLD construction prepared with MODELLER and displayed by VMD 1.8.6 [75]. The polyprotein encoded by the pFS-2 vector also contains a random coil region (a fragment of titin N2B, in grey) that acts as a spacer to avoid the noisy proximal region of the force-extension recordings, a major problem in AFM-SMFS [77]. The structural model was generated by aligning the ends of multiple ubiquitin structures (in black, PDB code 1UBQ), and the off-template generated structures for N2B (grey) and Orb2A PLD (dark yellow). (B) Representative force-extension recordings of pFS-2+Orb2A PLD using the “protection strategy” based on the pFS-2 vector [77]. This approach revealed different conformations adopted by the Orb2A PLD, from the NM (in yellow, left column) or M (in red, right column) conformations, showing different F and ΔLc values, and suggesting rich conformational polymorphism of this PLD at the monomer level. The elasticity of the stretched proteins was analyzed using the WLC model of polymer elasticity [78]. The carrier module (in grey) must unfold completely (“a” is ΔLc for it) before the force can access the guest Orb2A PLD: “b” and “c” represent the ΔLc for NM and M regions of Orb2A PLD, respectively; and b + c corresponds to the complete length of the stretched Orb2A PLD. The complete extension of Orb2A PLD is 64.8 nm (162 residues x 0.4 nm/residue), and that of I27 is 29.5 nm. (TIF) [file pbio.1002361.s008.tif]

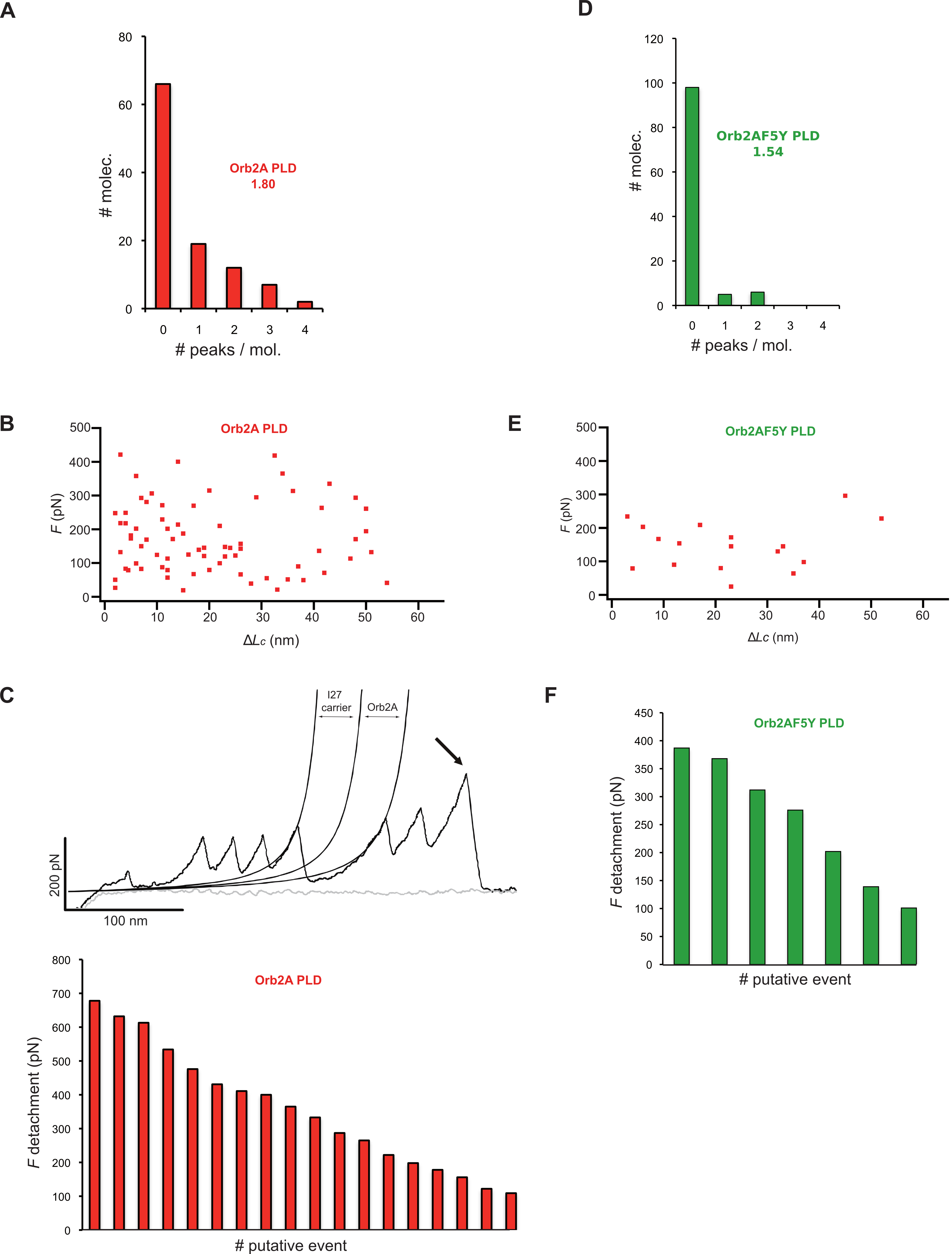

Supplement: S8 Fig — (A) The average number of force peaks (structures) per molecule is shown, where 0 represents the NM conformations. The M conformers usually show more than one force peak per Orb2A PLD molecule, suggesting the formation of several mechanical barriers at different positions of the molecule. (B) Scatter plots show that, in the M conformers, there is no clustering between F and ΔLc, which suggests that no preferred structures are formed. (C) The recording (top) represents an example of an incomplete or “putative” M event, in which we assume that the polyprotein attachments (either from the tip or the substrate) were lost before the molecule was fully stretched. Therefore, it is reasonable to assume that its F value would be higher than the detachment force (indicated by the arrow). The spectrum shows the force peaks due to the unfolding of the I27 carrier and a region of the Orb2A PLD that is shorter than the expected full-length protein (61 nm versus 94.3 nm from I27-Orb2A PLD complete unfolding). F detachment versus # of putative events analysis (bottom) suggests that the population of M conformers actually observed in our experiments is an underestimation. The presence of putative data (i.e., incomplete force-extension runs) strongly indicates that the true population of M conformers is probably more abundant than that observed. Putative data were not included in our sample size (n). (D) The average number of force peaks (structures) per molecule is decreased in the F5Y variant. (E) No evidence for the preferential formation of specific structures was obtained by SMFS of pFS-2+Orb2AF5Y PLD. (F) Putative incomplete recordings are fewer and of lower mechanical stability in the presence of the F5Y mutation suggesting the formation of fewer, weaker M conformers. The underlying data for panels in this figure can be found in S1 Data. (TIF) [file pbio.1002361.s009.tif]

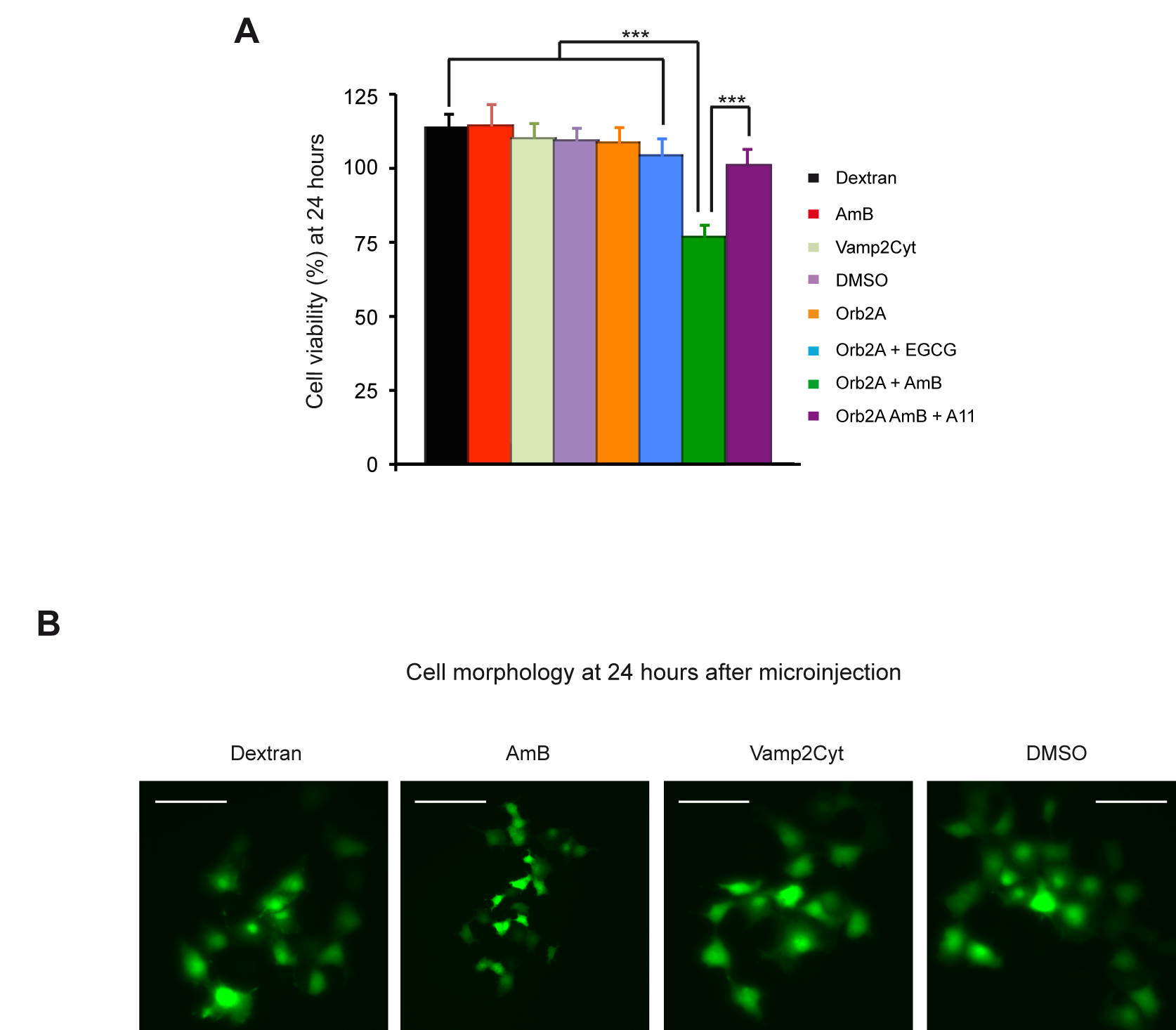

Supplement: S9 Fig — (A) Negative controls do not cause cell death after microinjection. (B) Survival of COS-7 cells microinjected with several samples after 24 h (***p < 0.001, one-way ANOVA and Tukey post-test). The underlying data for panels in this figure can be found in S1 Data. (TIF) [file pbio.1002361.s010.tif]

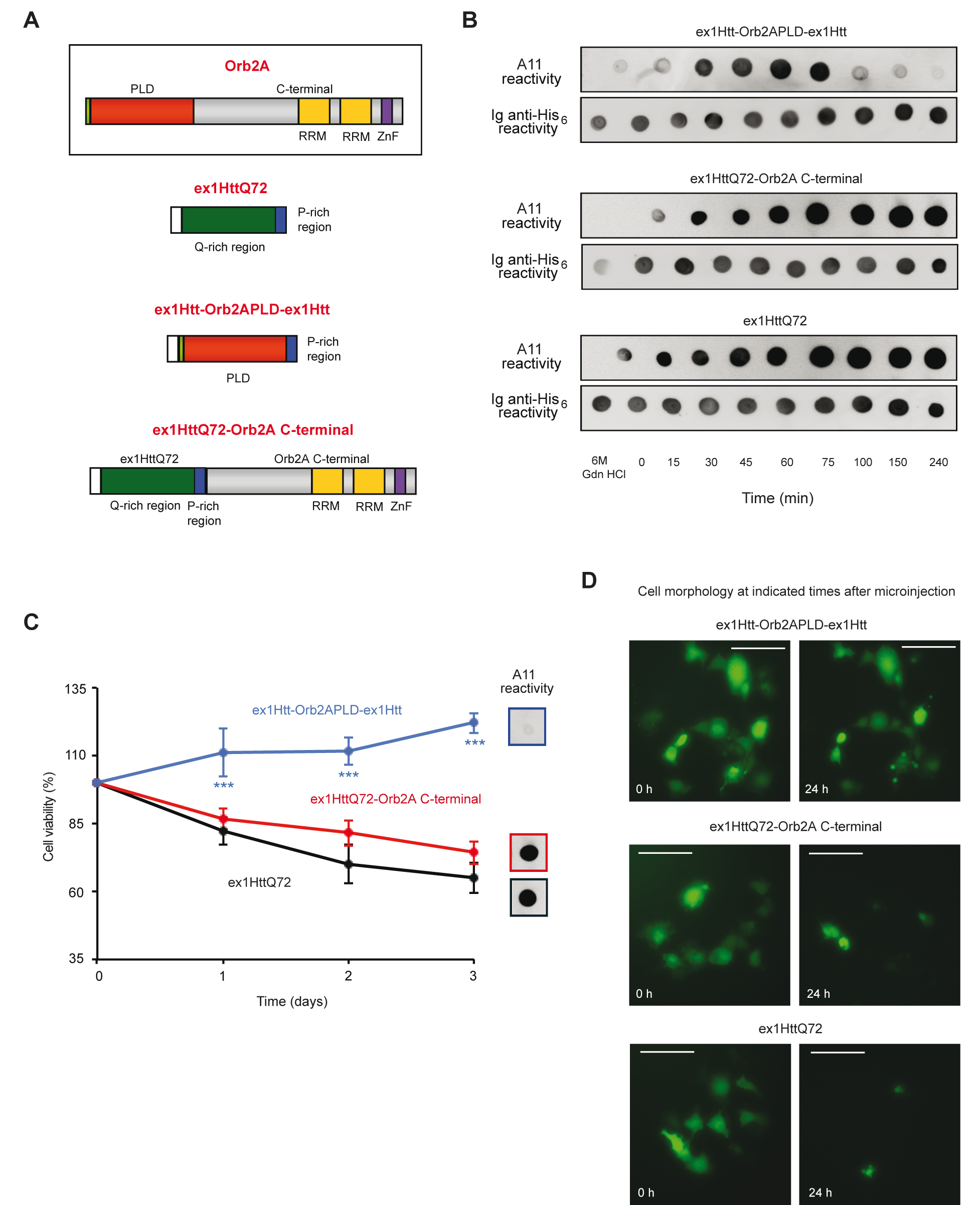

Supplement: S10 Fig — (A) Pictograms showing the domain organization of Orb2A, ex1HttQ72, and the chimeric proteins. (B) Structural features during the assembly of expanded exon 1 of Huntingtin (ex1HttQ72) and the chimeric constructions (ex1Htt-Orb2APLD-ex1Htt and ex1HttQ72-Orb2A C-terminal) probed with the conformational antibody A11 showed the formation of more stable species reactive to A11 for ex1HttQ72 and ex1HttQ72-Orb2A C-terminal. (C) Survival curves of COS-7 cells microinjected with the chimeric proteins (their A11-reactivity at microinjection time is indicated in an inset). Data are represented as the mean ± SEM: ***p < 0.001 (blue asterisks, ex1Htt-Orb2APLD-ex1Htt versus ex1HttQ72 and ex1HttQ72-Orb2A C-terminal; Two-way ANOVA and Bonferroni post-test). The number of single-cells microinjected per sample was n = 100–200. (D) Fluorescence micrographs of COS-7 cells microinjected with ex1HttQ72, ex1Htt-Orb2APLD-ex1Htt, and ex1HttQ72-Orb2A C-terminal at 0 and 24 h after microinjection. Microinjection of ex1HttQ72 and ex1HttQ72-Orb2A C-terminal resulted in a marked drop in the number of live cells at 24 h compared to those at 0 h, while ex1Htt-Orb2APLD-ex1Htt did not exhibit cytotoxicity. Scale bars: 100 μm. The underlying data for panels in this figure can be found in S1 Data. (TIF) [file pbio.1002361.s011.tif]

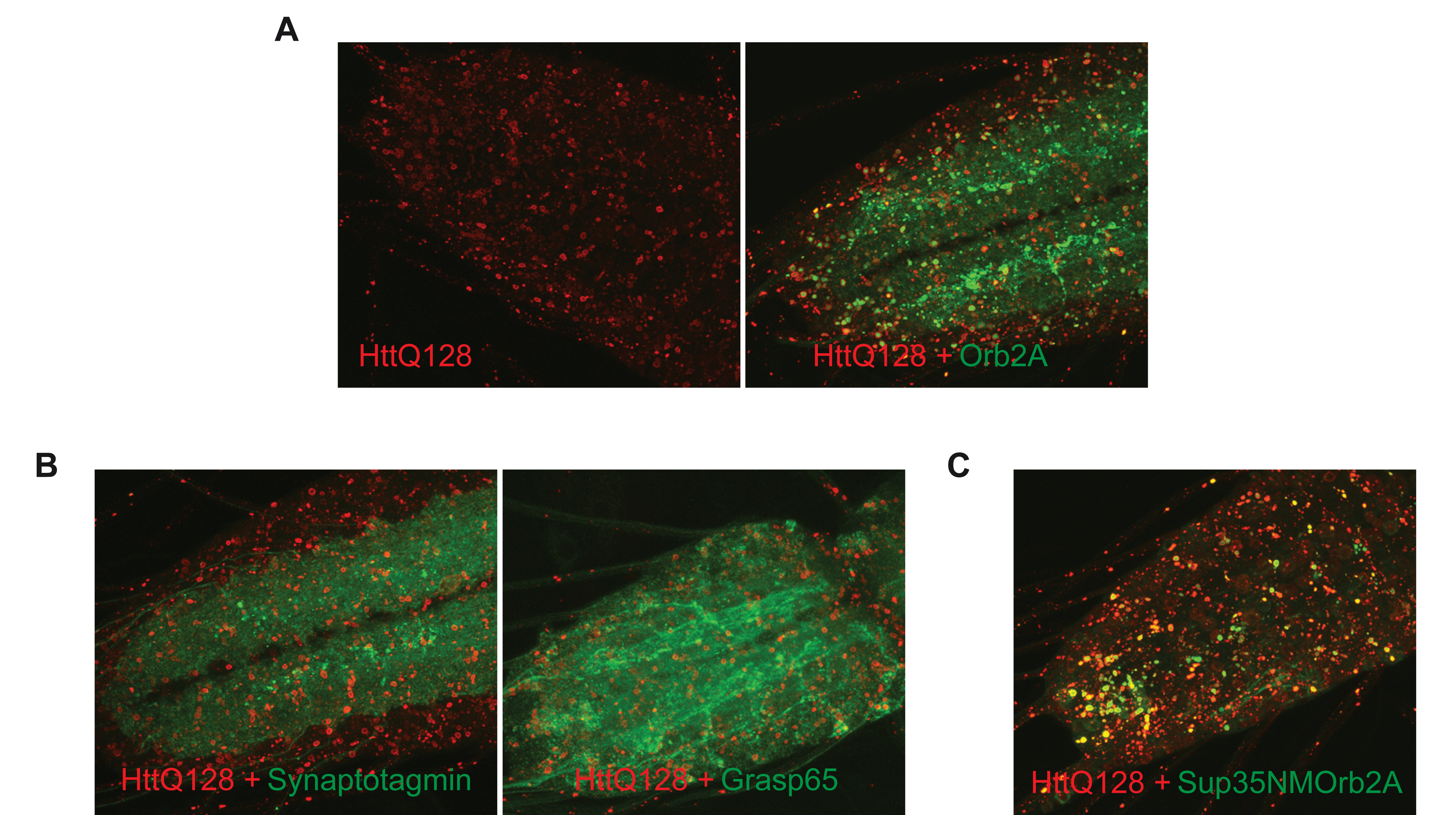

Supplement: S11 Fig — (A) HttQ128 forms larger fluorescence puncta that specifically recruit Orb2A to hetero-aggregates, whereas synaptic proteins like synaptotagmin or GRASP-65 are not recruited (B). (C) Replacing the Orb2A PLD with Sup35NM results in the formation of HttQ-Sup35NMOrb2AC hybrid oligomers. Scale bar: 20 μm. (TIF) [file pbio.1002361.s012.tif]

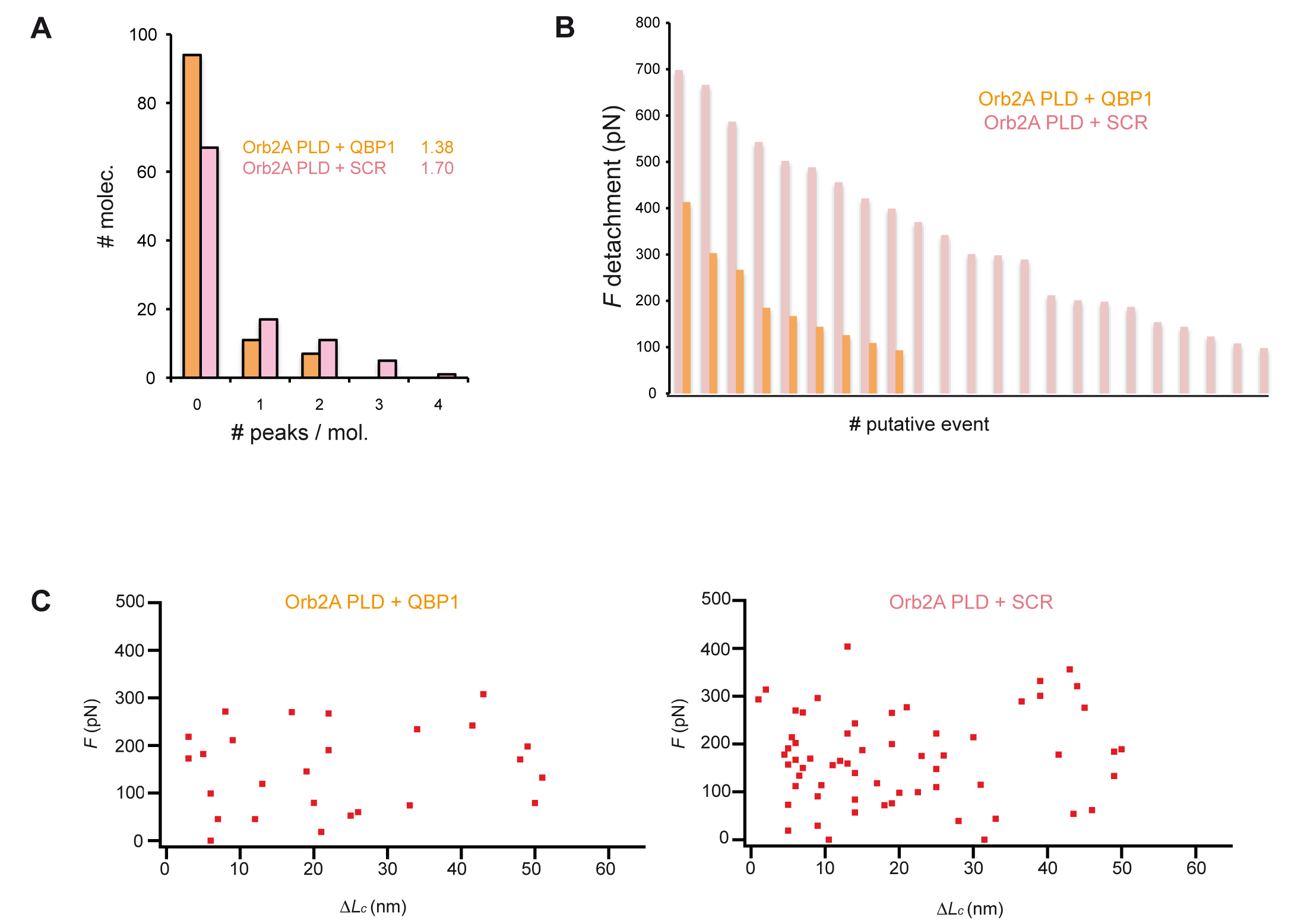

Supplement: S12 Fig — (A) The QBP1 peptide reduces the number of force peaks per molecule, suggesting that it decreases the propensity of Orb2A PLD to adopt β-structures (numbers in the inset are the average number of force peaks per Orb2A molecule). (B) Incomplete recordings of putative M events decrease in the presence of QBP1 inhibitor peptide, which suggests the formation of fewer, and weaker, M conformers. (C) No preferential structures were detected when stretching pFS-2+Orb2A PLD in the presence of QBP1 (left panel) or the SCR (right panel). The underlying data for panels in this figure can be found in S1 Data. (TIF) [file pbio.1002361.s013.tif]

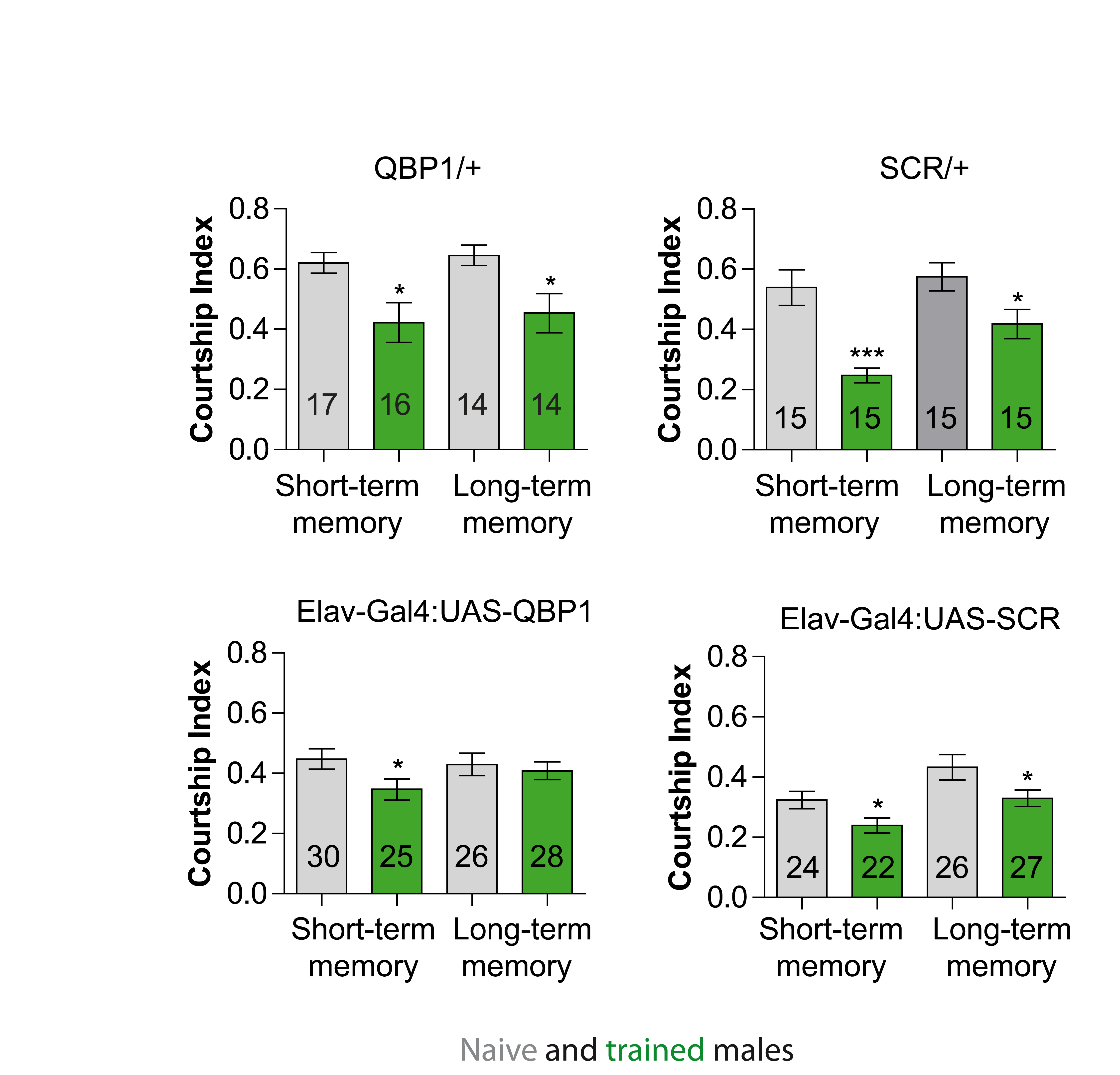

Supplement: S13 Fig — CI of male flies that were exposed to an unreceptive female was compared to that of naïve, untrained male flies. Pan-neuronal expression of QBP1 using the Elav-Gal4 driver interferes with long-term but not short-term memory, while the SCR control peptide has no effect (see Fig 7). The genetic controls for each group were tested simultaneously, and the number of flies used in each group is indicated. The underlying data for panels in this figure can be found in S1 Data. (TIF) [file pbio.1002361.s014.tif]

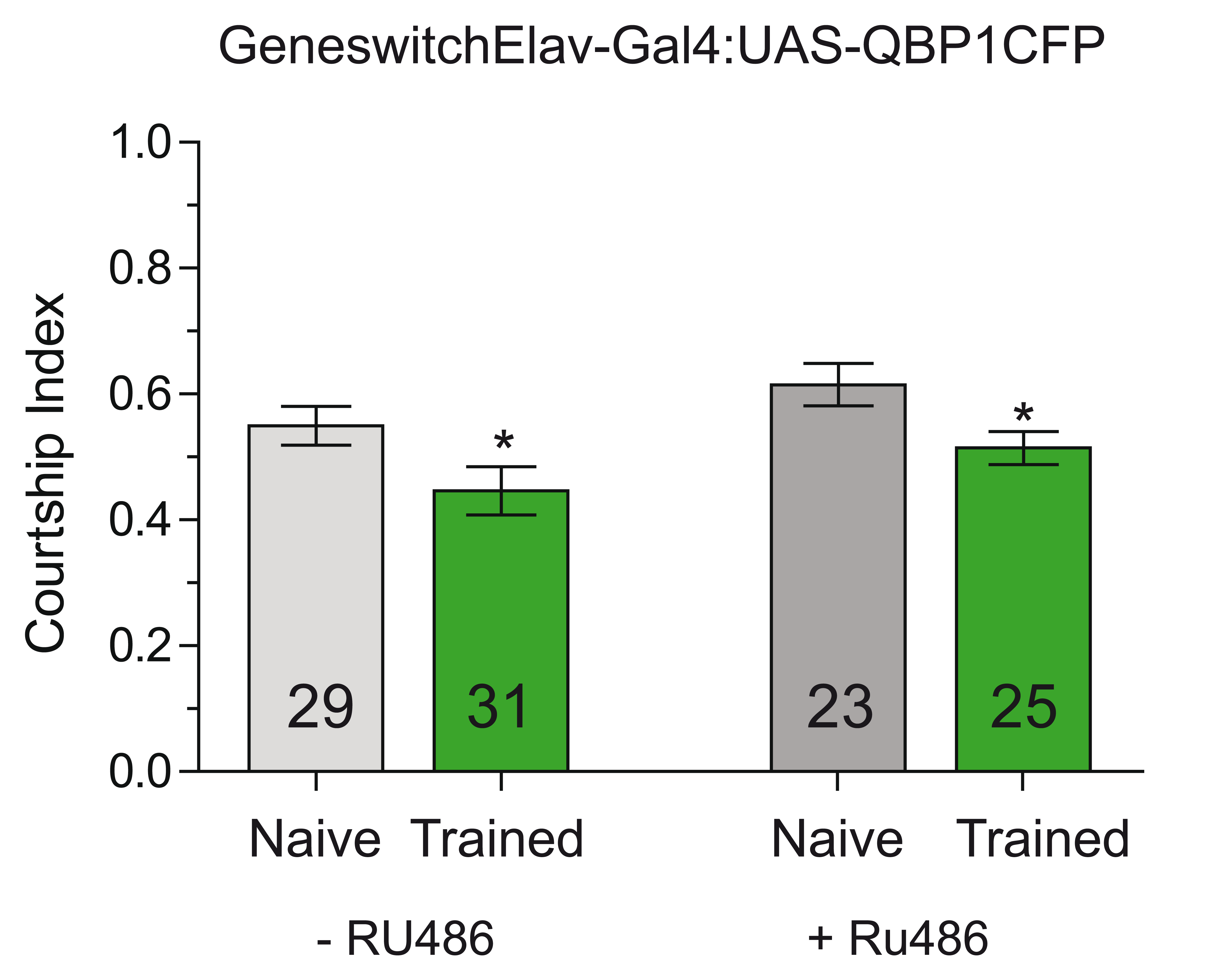

Supplement: S14 Fig — The QBP1 peptide was expressed in the adult nervous system 1 d prior to the behavioral training using the RU486-inducible GeneSwitch-Gal4 system. The 3–4 d old flies were fed with 1 mM RU486 for 24 h to induce the expression of QBP1. Following drug feeding the flies were trained in the male courtship suppression paradigm and memory was measured at 24 h. Both control (-RU486) and experimental groups showed significant courtship suppression at 24 h, suggesting that long-term memory was not affected by the transient expression of QBP1. The number of flies tested in various groups are indicated. *p < 0.05 (unpaired two-tailed Student’s t test). The underlying data for panels in this figure can be found in S1 Data. (TIF) [file pbio.1002361.s015.tif]

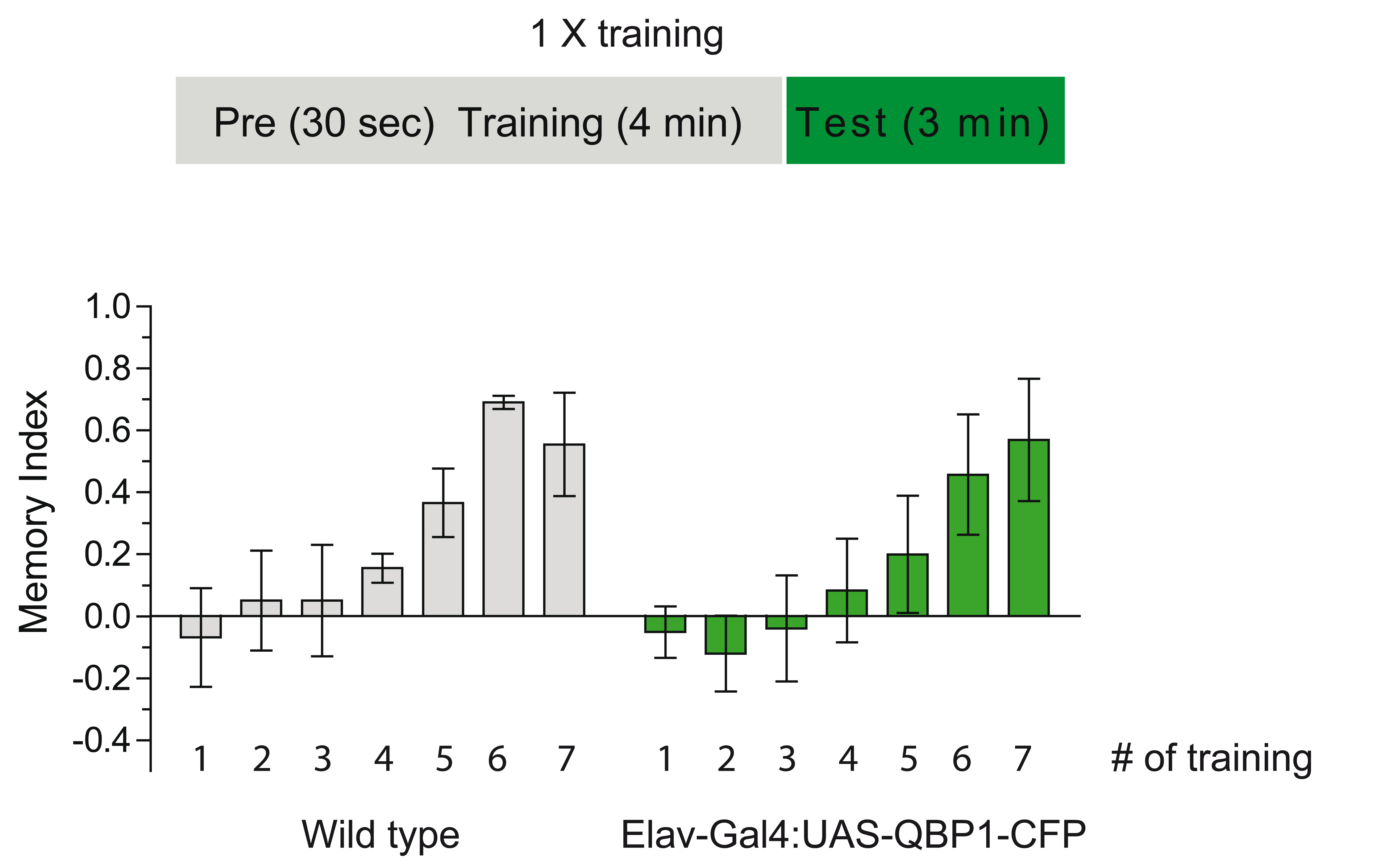

Supplement: S15 Fig — Top. Schematic of the single training protocol in the heat box operant conditioning paradigm. In this operant conditioning paradigm, individual flies were conditioned to avoid one side of a uniform chamber [74]. Each time the fly enters the predetermined “punish” side of the chamber, the temperature of the entire chamber is heated to 37°C and the temperature is brought back to 24°C when the fly moves to the other “unpunished” side. Following training, the flies learn to avoid the punish side even in the absence of punishment. Memory is measured as the duration of the preferred response for unpunished side. Flies are either trained in a single trial protocol or multiple trial protocol where the single training protocol is repeated successively the indicted times. Bottom. The avoidance index after each training session. With repeated training, the avoidance index increases, and after six training sessions the performance plateaus. There was no significant difference in the performance between wild type flies and flies expressing QBP1 chronically in the nervous system. The underlying data for panels in this figure can be found in S1 Data. (TIF) [file pbio.1002361.s016.tif]

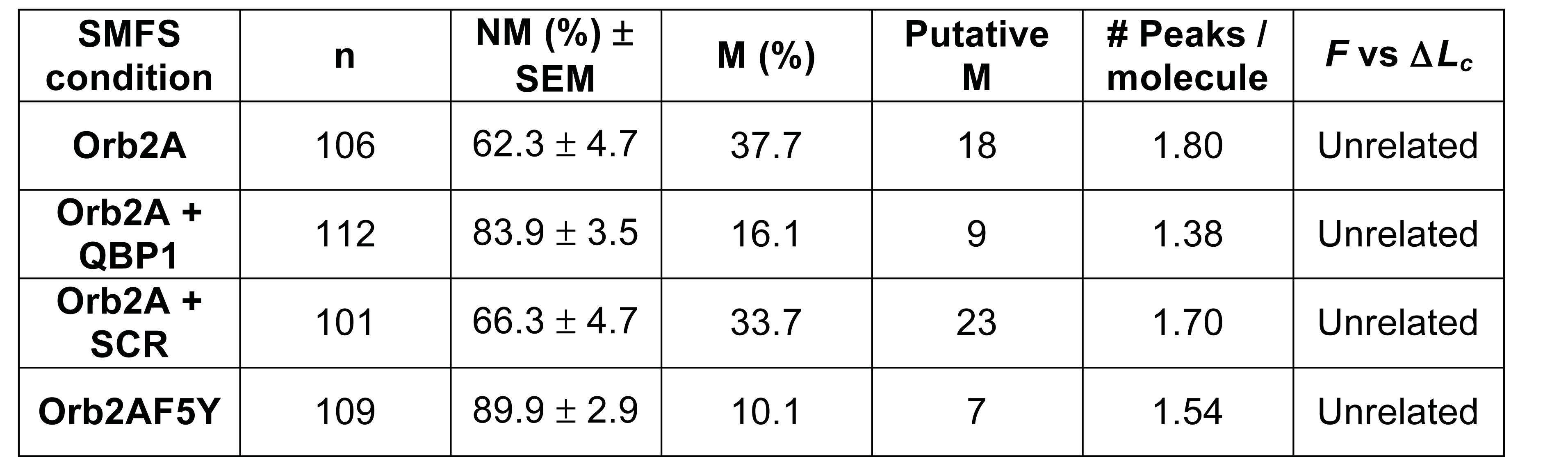

Supplement: S1 Table — NM, Nonmechanostable. M, mechanostable. SEM was calculated assuming the M and NM as states from a Bernoulli variable. The values are only shown in the NM column since they are the same as for the M states. Putative data were not included in our sample size (n). (TIF) [file pbio.1002361.s017.tif]

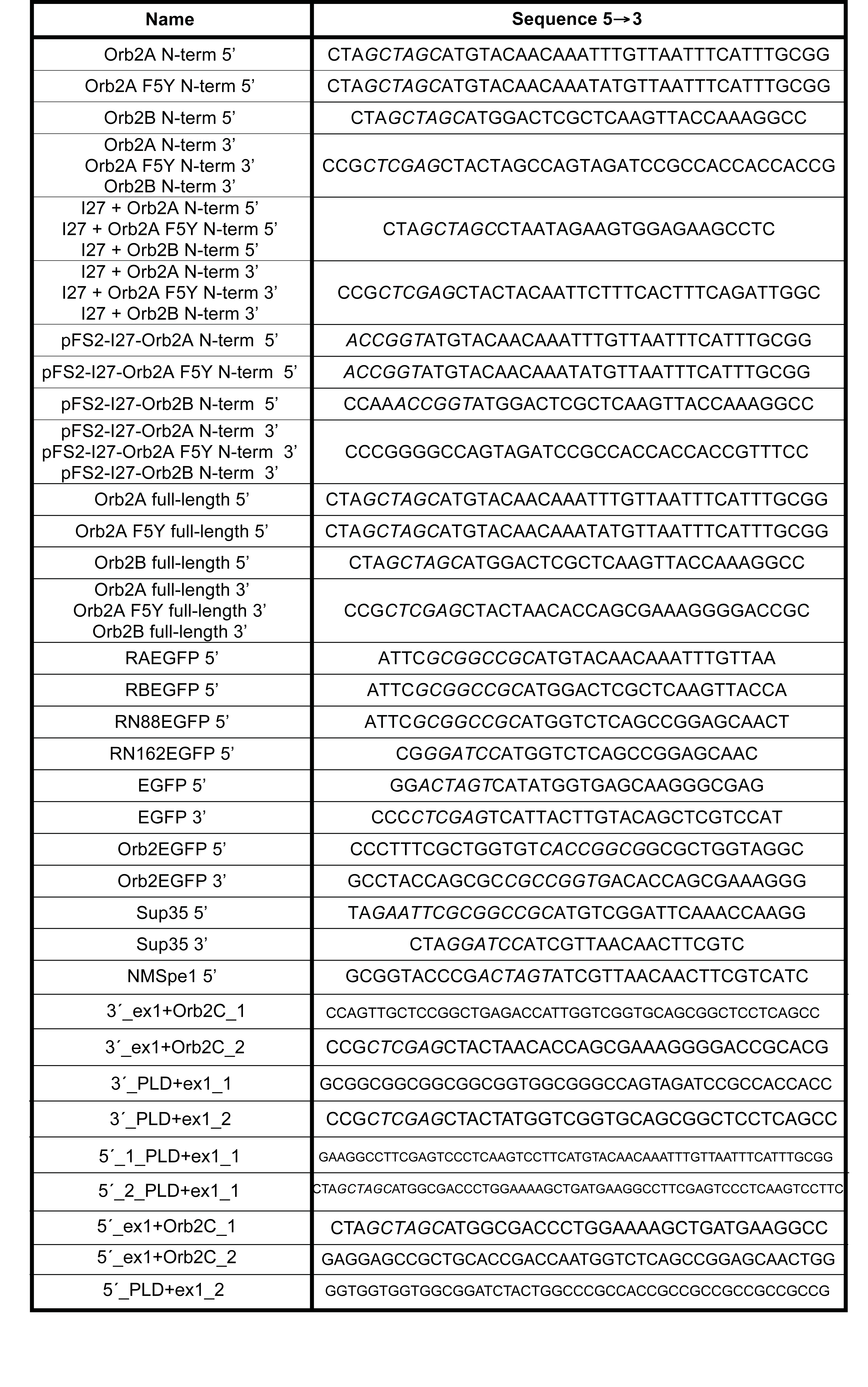

Supplement: S2 Table — The restriction sites introduced by PCR into the amplified sequences are in italics. The extra sequences added to each restriction site were recommended by New England Biolabs to enhance the digestion efficiency of linear DNA sequences. The vectors and strategy used are the same as those described previously [41]. (TIF) [file pbio.1002361.s018.tif]
